# Investigating the causal effects of smoking, sleep, and BMI on major depressive disorder and bipolar disorder: a univariable and multivariable two-sample Mendelian randomization study

**DOI:** 10.3389/fpsyt.2023.1206657

**Published:** 2023-10-12

**Authors:** Menglin He, Jian Zhou, Xuehan Li, Rurong Wang

**Affiliations:** Department of Anesthesiology, West China Hospital, Sichuan University, Chengdu, China

**Keywords:** life's essentials, major depressive disease, bipolar disorder, Mendelian randomization, causal effect

## Abstract

**Background:**

Mental disorders, characterized as products of biopsychosocial interactions, have emerged as a leading contributor to the worldwide rise in overall morbidity and disability rates. Life's essentials can affect nearly every aspect of our lives, from physical to mental health. In this study, we try to identify the associations between life's essentials and mental disorders.

**Method:**

Three assumptions of Mendelian randomization (MR) were applied to obtain the genetic instruments associated with smoking, sleep, and body mass index (BMI) in genome-wide association studies. Then, we conducted univariable MR (UVMR) and multivariable MR (MVMR) two-sample analyses to estimate the causal effects of these life's essentials on two mental disorders namely, major depressive disorder (MDD) and bipolar disorder (BD). Additionally, multiple sensitivity analyses were performed to evaluate the reliability and stability of the study results.

**Results:**

In the MR analysis of the association of smoking, sleep, and BMI with MDD, we obtained 78, 39, and 302 genetic instruments, respectively. Smoking [odds ratio (OR), 1.03; 95% confidence interval (CI), 1.01–1.06; *p* = 0.004], sleep (OR, 1.04; 95% CI, 1.02–1.06; *p* < 0.001), and BMI (OR, 1.01; 95% CI, 1.01–1.02; *p* < 0.001) were all considered as risk factors for MDD and were independent of each other (smoking: OR, 1.03, 95% CI, 1.01–1.06, *p* = 0.008; sleep: OR, 1.03, 95% CI, 1.01–1.05, *p* = 0.001; and BMI: OR, 1.01, 95% CI, 1.01–1.02, *p* < 0.001). Additionally, 78, 38, and 297 genetic instruments were obtained in the MR analysis of smoking, sleep, and BMI with BD, respectively. Causal associations were observed between smoking (OR, 2.46; 95% CI, 1.17–5.15; *p* = 0.017), sleep (OR, 2.73; 95% CI, 1.52–4.92; *p* < 0.001), and BD, and smoking (OR, 2.43; 95% CI, 1.69–3.16; *p* = 0.018) might be a mediator in the causal effects of sleep on BD. Finally, there was no inconsistency between sensitivity and causality analysis, proving that our results are convincing.

**Conclusion:**

The study results provide strong evidence that smoking, sleep, and BMI are causally related to MDD and BD, which need further research to clarify the underlying mechanism.

## 1. Introduction

The WHO defines mental health as “a state of sustained and positive development of the mind in which people are well-adapted and fully reach their physical and mental potential” (1). However, approximately one-third of people suffer from mental disorders in their lifetime, and even this statistic might be underestimated (2). Extensive research has been conducted to explore the pathogenesis of mental disorders and related pharmacological treatments but with limited results (3, 4). People affected by mental illnesses are heavily neglected because of the financial burden of the disease, especially in developing countries (5). Therefore, low-cost and high-efficiency measures to prevent disease burden in individuals by identifying risk factors for mental health are urgently needed.

Life's essentials, including a healthy diet, participation in physical activity, avoidance of nicotine, healthy sleep, healthy weight, and healthy levels of blood lipids, blood glucose, and blood pressure, constitute a novel framework defined by the American Heart Association to help doctors shift their focus from solely treating diseases to promoting positive psychological health (6). Therefore, determining the effects of life's essentials on mental disorders might provide new insights into this therapeutic field. In this study, we focused on three of life's eight essentials, namely, smoking, sleep, and body mass index (BMI). Previous studies have indicated a strong relationship between smoking and mental disorders, with the severity of the illness increasing with the increase in smoking rate (7). Moreover, reducing smoking initiation appears to be an effective strategy for preventing psychiatric disorders, although its impact may be limited among patients with depression (8). Another meta-analysis found a close association between smoking and depression, with smoking increasing the risk of depression by 1.73 times compared to non-smoking (9). Additionally, aberrant sleep patterns could potentially serve as valuable markers for psychiatric disorders, offering promising opportunities for both prevention and management of these conditions (10, 11). Furthermore, Mendelian randomization (MR) studies have provided evidence supporting the causal relationship between obesity and an elevated risk of depression, and the rising obesity rates may have contributed, to some extent, to the increased prevalence of depressive symptoms (12). Although randomized clinical trials have illustrated the effects of smoking, sleep, and BMI on diseases, more accurate and scientific studies are needed to disentangle these associations without the interference of conventional clinical trials, which could be affected by residual confounding and reverse causality.

MR is a recently developed statistical tool that uses genetic variants as instrumental variables (IVs) to estimate the causal effect of exposure on the outcome without any bias from residual confounding or reverse causality (13). Moreover, MR permits unrestricted exchanges between exposures and outcomes, greatly enhancing its practical value. MR is based on the law of independent assortment: genes on non-homologous chromosomes tend to combine freely while alleles are separated in the offspring generation to produce gametes (14). Furthermore, genome-wide association studies (GWAS) greatly contribute to obtaining reliable and stable MR results.

We performed univariable MR (UVMR) and multivariable MR (MVMR) analyses to estimate the causal effects of life's essentials on mental disorders. In these analyses, smoking, sleep, and BMI were considered as exposures, and major depressive disorder (MDD) and bipolar disorder (BD) were considered as outcomes.

## 2. Materials and methods

### 2.1. Study design and data sources

**Figure 1:**
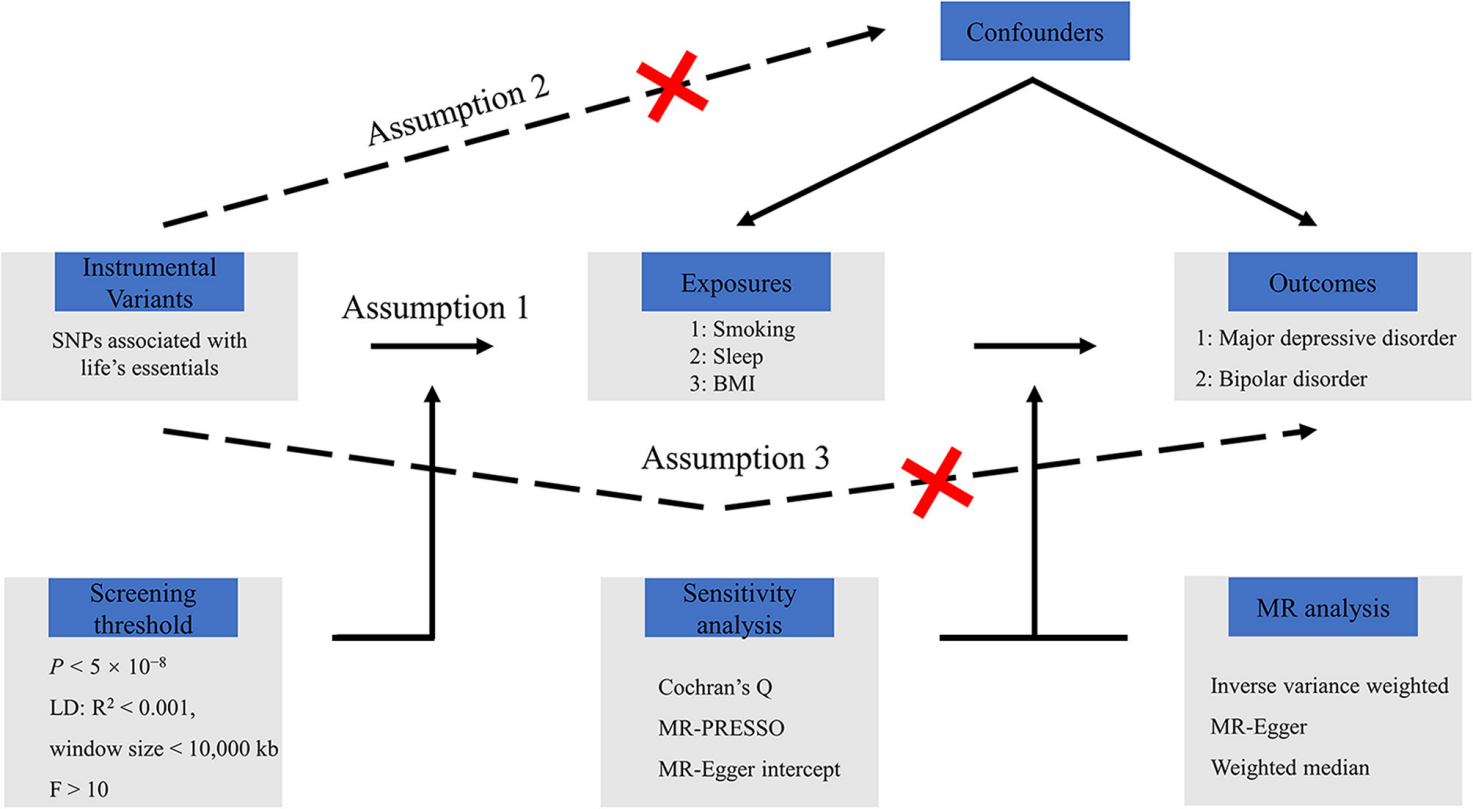
Flowchart of Mendelian randomization study.

The flowchart for UVMR and MVMR analyses is shown in Figure 1. Three assumptions of MR were applied to analyze correlation, exclusivity, and independence between exposures and outcomes (15). First, there is a close association between genetic IVs and exposures. Second, genetic IVs exert effects on outcomes only through exposure. Finally, the interference of confounding factors in genetic IVs is non-existent. The IEU GWAS database (https://gwas.mrcieu.ac.uk), a publicly accessible online tool containing GWAS summary data, was used to obtain data on three of life's eight essentials and two mental disorders. GWAS of exposure 1 (ever smoked, https://gwas.mrcieu.ac.uk/datasets/ukb-b-20261/) included 280,508 cases and 180,558 control individuals; GWAS of exposure 2 (sleeplessness/insomnia, https://gwas.mrcieu.ac.uk/datasets/ukb-b-3957/) included 462,341 samples; and GWAS of exposure 3 (BMI, https://gwas.mrcieu.ac.uk/datasets/ukb-a-248/) included 336,107 samples. Additionally, GWAS of outcome 1 (MDD, https://gwas.mrcieu.ac.uk/datasets/ebi-a-GCST005903/) included 8,276 cases and 209,308 control individuals. GWAS of outcome 2 (BD, https://gwas.mrcieu.ac.uk/datasets/ieu-b-41/) included 20,352 cases and 31,358 control individuals. GWAS data for exposures 1 and 2 were obtained from the MRC-IEU consortium, and exposure 3 data were collected from Neale Lab. GWAS data for outcome 2 were gathered from the Bipolar Disorder Working Group of the Psychiatric Genomics Consortium. The GWAS data employed in this study had been approved by the relevant ethics committees with informed consent from participants.

### 2.2. Selection of genetic IVs

To meet the three assumptions of MR analysis, *p* < 5 × 10^−8^ was set as an association threshold to select eligible single nucleotide polymorphisms (SNPs). We used the clumping method among the selected SNPs with R^2^ < 0.001 and physical distance < 10,000 kb to avoid linkage disequilibrium (LD). In addition, for SNPs representing incompatible alleles or with palindromic characteristics and intermediate allele frequencies, we employed the SNP harmonization method to harmonize their effects (16). SNPs with a minor allele frequency <0.01 were excluded. Finally, we used the F statistics (*F* = β^2^/se^2^) to evaluate the power of the test of the remaining SNPs and removed SNPs with weak power (*F* statistics < 10) (17).

### 2.3. MR analysis

In UVMR analysis, inverse variance weighted (IVW) (18), MR-Egger (19), and weighted median (20) methods were used to assess the causal effects of exposures (smoking, sleeplessness/insomnia, and BMI) on outcomes (MDD and BD). IVW, as the standard method for summarizing MR data, directly calculates the causal effect values using summary statistics rather than individual statistics. MR-Egger, while possessing weaker power than IVW due to its assumption of independence between IV-exposure and IV-outcome associations, addresses pleiotropy between genetic variants. A weighted median requires that at least 50% of the weights attributed to genetic variations are valid. Consequently, each method exhibits its own strengths and limitations concerning the consistency of causal effect estimation and the test power. We further performed the MVMR analysis, an extension of UVMR, to determine the independent risk factors in multiple exposures (21), and used the IVW model as the primary assessment method.

### 2.4. Sensitivity analysis

Sensitivity analysis is an essential part of MR analysis and includes heterogeneity and pleiotropy analyses. We employed Cochran's Q statistics, along with IVW and MR-Egger regression, to test the heterogeneity of IVs (22). In addition, the MR Pleiotropy RESidual Sum and Outlier (MR-PRESSO) test was conducted to detect horizontal pleiotropy in IVs (23). This comprehensive test comprises the MR-PRESSO global test, which assesses the presence of horizontal pleiotropy; the MR-PRESSO outlier test, which estimates the corrected results after outlier removal; and the MR-PRESSO distortion test, which scrutinizes any disparities between pre-correction and post-correction results. IVs with a positive result in the MR-PRESSO distortion test were removed. Besides, the MR-Egger intercept could also identify and correct horizontal pleiotropy (24). A leave-one-out analysis was conducted to assess whether the causal effect of exposures on outcomes was biased by any single SNP to further validate the results. All statistical analyses and data visualizations were conducted using the R packages “Two sample MR packages,” “Mendelian randomization,” and “MVMR” in the R software version 4.1.2. Results with *p* < 0.05 were considered statistically significant.

## 3. Results

### 3.1. Identification of IVs for MR analysis

Following the comprehensive and rigorous selection process, a total of 78, 39, and 302 SNPs were identified as IVs in the causal effect analysis of the association between smoking, sleeplessness/insomnia, BMI, and MDD. Furthermore, 78, 38, and 297 SNPs were obtained for BD (Table 1). All SNPs had a strong power of test with F statistics greater than the accepted threshold of 10. Detailed information regarding these IVs is available in Supplementary Tables S1–S6.

**Table 1:** Characteristics of the included genome-wide association studies.

**Traits**	**Unit**	**Sample size**	**Population**	**Consortium**	**IVs (of MDD/BD)**
Ever smoked	SD	461,066	European	MRC-IEU	78/78
Sleeplessness/insomnia	SD	462,341	European	MRC-IEU	39/38
BMI	SD	336,107	European	Neale Lab	302/297
MDD	logOR	217,574	European	-	-
BD	-	51,710	European	BDWGPGC	-

### 3.2. UVMR analysis

First, we estimated the causal effects of smoking, sleeplessness/insomnia, and BMI on MDD using the IVW, MR-Egger, and weighted median methods. In the IVW analysis, the log odds ratio (logOR) of MDD increased 1.03-fold with one standard deviation (SD) increase in smoking [95% confidence interval (CI), 1.01–1.06, *p* = 4.10 × 10^−3^]. Similar results were obtained in the weighted median analysis (OR, 1.03, 95% CI, 1.00–1.07, *p* = 0.044). As for the MR-Egger test, smoking could be regarded as a risk factor, but the *p-*value was not significant (OR, 1.01, 95% CI, 0.91–1.11, *p* = 0.911). Furthermore, the logOR of MDD increased 1.04-fold with one SD increase in sleeplessness/insomnia in the IVW analysis (95% CI, 1.02–1.06, *p* = 7.06 × 10^−4^) and weighted median analysis (95% CI, 1.01–1.08, *p* = 1.83 × 10^−3^). The value of MR-Egger analysis was not statistically significant (OR, 1.06, 95% CI, 0.99–1.14, *p* = 0.125). As for BMI, both IVW (OR, 1.01, 95% CI, 1.01–1.02, *p* = 6.04 × 10^−11^) and weighted median (OR, 1.01, 95% CI, 1.01–1.02, *p* = 1.80 × 10^−5^) analyses showed the existence of causal effects between BMI and MDD, while the MR-Egger analysis found no significant causal effect (OR, 1.01, 95% CI, 1.00–1.02, *p* = 0.232) (Table 2). The results of Cochran's Q test showed that there was no heterogeneity between smoking and MDD (Q = 90.55, *p* = 0.139), while heterogeneity existed between sleeplessness/insomnia, BMI, and MDD (Q = 57.88, *p* = 0.02; Q = 366.59, *p* = 5.75 × 10^−3^). MR-PRESSO and MR-Egger intercept tests did not find pleiotropy in IVs of smoking (*p* = 0.143; intercept = 1.86 × 10^−4^, *p* = 0.623), while there was pleiotropy in IVs of sleeplessness/insomnia (*p* = 0.029; intercept = 2.27 × 10^−4^, *p* = 0.591) and BMI (*p* = 0.007; intercept = 1.36 × 10^−4^, *p* = 0.267); in the MR-PRESSO distortion test, BMI had a *p*-value of 0.802, indicating that there was no significant difference between the results before and after outlier correction (Table 3). A leave-one-out analysis was conducted to test whether a single SNP could strongly reverse the causal effect; none of the IVs significantly altered the extent of causation between exposures and outcomes, indicating that the results were reliable (Supplementary Figure S1).

**Table 2:** MR results for the causal effect of three life's essentials and two mental disorders.

**Ever smoked**		
**MDD**	**OR (95% CI)**	***p***
Inverse variance weighted	1.03 (1.01, 1.06)	0.004
MR-Egger	1.01 (0.90, 1.12)	0.911
Weighted median	1.03 (1.00, 1.07)	0.044
Multivariable	1.03 (1.01, 1.06)	0.008
**BD**		
Inverse variance weighted	2.46 (1.17, 5.15)	0.017
MR-Egger	7.02 (0.20, 251.67)	0.289
Weighted median	3.45 (1.50, 7.93)	0.004
Multivariable	2.43 (1.69, 3.16)	0.018
**Sleeplessness/insomnia**		
**MDD**		
Inverse variance weighted	1.04 (1.02, 1.06)	< 0.001
MR-Egger	1.06 (0.99, 1.14)	0.125
Weighted median	1.04 (1.01, 1.08)	0.002
Multivariable	1.03 (1.01, 1.05)	0.001
**BD**		
Inverse variance weighted	2.73 (1.52, 4.92)	< 0.001
MR-Egger	6.72 (1.01, 44.86)	0.057
Weighted median	2.15 (1.08, 4.26)	0.029
Multivariable	1.43 (0.81, 2.04)	0.257
**BMI**		
**MDD**		
Inverse variance weighted	1.01 (1.01, 1.02)	< 0.001
MR-Egger	1.01 (0.995, 1.019)	0.232
Weighted median	1.01 (1.01, 1.02)	< 0.001
Multivariable	1.01 (1.01, 1.02)	< 0.001
**BD**		
Inverse variance weighted	0.93 (0.83, 1.05)	0.223
MR-Egger	1.14 (0.81, 1.61)	0.45
Weighted median	1.03 (0.88, 1.20)	0.74
Multivariable	0.90 (0.77, 1.03)	0.12

OR, odds ratio; CI, confidence interval.

**Table 3:** MR results for the sensitivity analyses of three life's essentials and two mental disorders.

	**IVW**	**MR-Egger**		**MR-PRESSO**		**MR-Egger intercept**	
	***p***	***p***	*p*_globaltest_	*p*_outliertest_	*p*_distortiontest_	**Intercept**	***p***
**MDD**							
Ever smoked	0.139	0.126	0.143	-	-	< 0.001	0.623
Sleeplessness/Insomnia	0.020	0.017	0.029	-	-	< -0.001	0.591
BMI	0.006	0.006	0.007	4.22 × 10^−10^	0.802	< 0.001	0.267
**BD**							
Ever smoked	3.70 × 10^−11^	3.02 × 10^−11^	< 0.001	0.006	0.746	−0.007	0.559
Sleeplessness/Insomnia	0.001	0.001	0.001	< 0.001	0.741	−0.011	0.335
BMI	9.72 × 10^−19^	1.42 × 10^−18^	< 0.001	0.256	0.861	−0.004	0.214

MR, Mendelian randomization; MR-PRESSO, MR-pleiotropy residual sum and outlier method.

The same analyses were performed to investigate the causal effects of smoking, sleeplessness/insomnia, and BMI on BD. Both IVW (OR, 2.46, 95% CI, 1.18–5.15, *p* = 0.017) and weighted median (OR, 3.45, 95% CI, 1.50–7.93, *p* = 3.62 × 10^−4^) identified smoking as a risk factor for BD, while the result of MR-Egger analysis was not significant (OR, 7.02, 95% CI, 0.20–251.666, *p* = 0.289). As for sleeplessness/insomnia, IVW (OR, 2.733, 95% CI, 1.518–4.922, *p* = 8.09 × 10^−4^), weighted median (OR, 2.15, 95% CI, 1.08–4.26, *p* = 0.029), and MR-Egger (OR, 6.72, 95% CI, 1.01–44.86, *p* = 0.057) analyses were conducted, indicating a causal relationship between sleeplessness/insomnia and BD. Regarding BMI, none of the methods revealed a causal effect: IVW (OR, 0.93, 95% CI, 0.83–1.05, *p* = 0.224), weighted median (OR, 1.03, 95% CI, 0.88–1.20, *p* = 0.739), and MR-Egger (OR, 1.14, 95% CI, 0.81–1.61, *p* = 0.450) (Table 2). In Cochran's Q test, heterogeneity was observed in smoking (Q = 187.16, *p* = 3.70 × 10^−11^), sleeplessness/insomnia (Q = 69.31, *p* = 1.01 × 10^−3^), and BMI (Q = 562.16, *p* = 9.72 × 10^−19^). The results of the MR-PRESSO global test indicated the existence of directional pleiotropy in IVs of smoking (*p* = 3.33 × 10^−4^), sleeplessness/insomnia (*p* = 0.001), BMI (p = 3.33 × 10^−4^), and BD, while the MR-PRESSO distortion test showed that outlier SNPs did not bias the results for smoking (*p* = 0.746), sleeplessness/insomnia (*p* = 0.741), and BMI (*p* = 0.861) (Table 3). The results of the leave-one-out analysis of BD were the same as those of MDD, except for BMI exposure (Supplementary Figure S1). The scatter plots, forest plots, and funnel plots were visualized using the R software (Supplementary Figures S2–S4).

### 3.3. MVMR analysis

In the MVMR analysis, strong evidence was found for the causal effects of smoking, sleeplessness/insomnia, and BMI on MDD based on the IVW method (smoking: OR, 1.03, 95% CI, 1.01–1.06, *p* = 8.29 × 10^−3^; sleeplessness/insomnia: OR, 1.03, 95% CI, 1.01–1.05, *p* = 1.03 × 10^−3^; and BMI: OR, 1.01, 95% CI, 1.01–1.02, *p* = 1.48 × 10^−7^), indicating these exposures might be independent risk factors for MDD. The results of the causal effects of these life's essentials on BD showed that smoking (OR, 2.43, 95% CI, 1.69–3.16, *p* = 0.018) might increase the risk of BD, while sleeplessness/insomnia (OR, 1.43, 95% CI, 0.81–2.04, *p* = 0.257) and BMI (OR, 0.90, 95% CI, 0.77–1.03, *p* = 0.119) were not significantly associated with BD (Table 2).

## 4. Discussion

The UVMR analysis indicated that smoking, sleeplessness/insomnia, and BMI are risk factors for MDD, and the MVMR analysis further confirmed their independence from each other. Furthermore, smoking and sleeplessness/insomnia were associated with an increased risk of BD, with the possibility of sleeplessness/insomnia affecting BD through smoking mediation. Conversely, no causal effect between BMI and BD was observed. These findings suggest that increasing attention should be paid to these three life's essentials in preventing MDD and BD.

The debate about whether mental disorders lead to smoking or whether smoking leads to mental disorders is ongoing (25). A meta-review identified smoking as a detrimental factor for several psychiatric conditions (26), aligning with our results. However, another hypothesis suggests that individuals who smoke may develop symptoms of mental disorders (27). Such inconsistency between the findings may be partially attributed to the limited definition of smoking in our study, which focused solely on smoking or non-smoking. A comprehensive analysis should encompass factors such as smoking onset, smoking status, heaviness of smoking, tobacco dependence, and smoking trajectory (7). The findings of this study can contribute to more comprehensive studies on the associations between smoking and mental disorders.

Adequate and high-quality sleep has been proposed as a potential factor in preventing mental disorders in both young and older adults (28, 29). Moreover, cognitive behavior therapy has shown significant reductions in depressive symptoms compared to control conditions (26). Similar results were obtained in our MR analyses. While a large prospective meta-analysis of data including 172,007 cases linked insomnia to an increased risk of depression (30), our present study did not find a causal effect of sleep on MDD. This inconsistency may stem from individuals' subjective feelings about sleep-related phenotypes due to the variety and ambiguity of sleep traits (31). More precise and objective MR analyses are needed to get accurate results in sleep-related studies.

Decades of research have illustrated the causal effect of BMI on cancer (32), cardiovascular disease (33), type 2 diabetes (34), and venous thromboembolism (35). However, studies related to mental disorders are limited. An authoritative systematic review acknowledged that the evidence supporting the decrease in BMI leading to the recovery of mental disorders was not convincing. Nonetheless, it is recommended that future studies should focus on lifestyle interventions, including diet and physical activity (36). Our results also showed that BMI is causally related to MDD but not BD. This conflicting phenomenon may be related to the potential mediating role of smoking in the causal effect of BMI on BD. As reported by a previous study, smoking could be a risk factor for increased BMI (37). Therefore, comprehensive MR analyses exploring the association between smoking and BMI are needed to support our results.

We should consider the study's limitations. First, providing more detailed information about ancestry subgroups in GWAS can help mitigate uncontrolled confounding from familial effects (38). In addition, pleiotropy, a common issue in MR analysis, can lead to result confusion (39). To prevent this, we used the MR-Egger and MR-PRESSO methods to evaluate pleiotropy, ensuring the clarity of our study results. Finally, the binary nature of smoking and sleep phenotypes in our MR analysis, without incorporating multiple subgroups, could potentially impact the accuracy of the results.

Nonetheless, this study possesses several strengths. First, to the best of our knowledge, this MR analysis is the first attempt to explore the causal effects of life's essentials on mental disorders, highlighting the significance of life's essentials in improving mental health. Second, we obtained adequate SNPs as IVs and used multiple analysis methods, which may be conducive to minimizing the effect of bias and obtaining robust results. Third, the MVMR analysis aimed to detect any independent risk factors for mental disorders among life's essentials.

In conclusion, this study provides strong evidence that life's essentials, such as smoking, sleep, and BMI, are causally related to MDD and BD. However, the causal relationship between BMI and BD needs further exploration.

## Data availability statement

The original contributions presented in the study are included in the article/Supplementary material, further inquiries can be directed to the corresponding authors.

## Ethics statement

Ethical review and approval was not required for the study on human participants in accordance with the local legislation and institutional requirements. Written informed consent from the patients/ participants or patients/participants' legal guardian/next of kin was not required to participate in this study in accordance with the national legislation and the institutional requirements.

## Author contributions

RW and XL designed this study. MH performed the statistical analysis of data. JZ conducted the draft of the manuscript. All authors read and approved the final manuscript.

## Supplementary material

Supplementary Figure S1 Leave-one-out plots of MR analyses for **(A)** smoking, **(B)** sleeplessness/insomnia, and **(C)** BMI on major depressive disease, and for **(D)** smoking, **(E)** sleeplessness/insomnia, and **(F)** BMI on bipolar disorder.

Supplementary Figure S2 Scatter plots of MR analyses for **(A)** smoking, **(B)** sleeplessness/insomnia, and **(C)** BMI on major depressive disease, and for **(D)** smoking, **(E)** sleeplessness/insomnia, and **(F)** BMI on bipolar disorder.

Supplementary Figure S3 Forest plots of MR analyses for **(A)** smoking, **(B)** sleeplessness/insomnia, and **(C)** BMI on major depressive disease, and for **(D)** smoking, **(E)** sleeplessness/insomnia, and **(F)** BMI on bipolar disorder.

Supplementary Figure S4 Funnel plots of MR analyses for **(A)** smoking, **(B)** sleeplessness/insomnia, and **(C)** BMI on major depressive disease, and for **(D)** smoking, **(E)** sleeplessness/insomnia, and **(F)** BMI on bipolar disorder.
