# Supplementary figures and images for: Investigating the causal effects of smoking, sleep, and BMI on major depressive disorder and bipolar disorder: a univariable and multivariable two-sample Mendelian randomization study
Source: Front Psychiatry. 2023 Oct 12;14:1206657. doi: 10.3389/fpsyt.2023.1206657 (PMC10602671; doi:10.3389/fpsyt.2023.1206657)

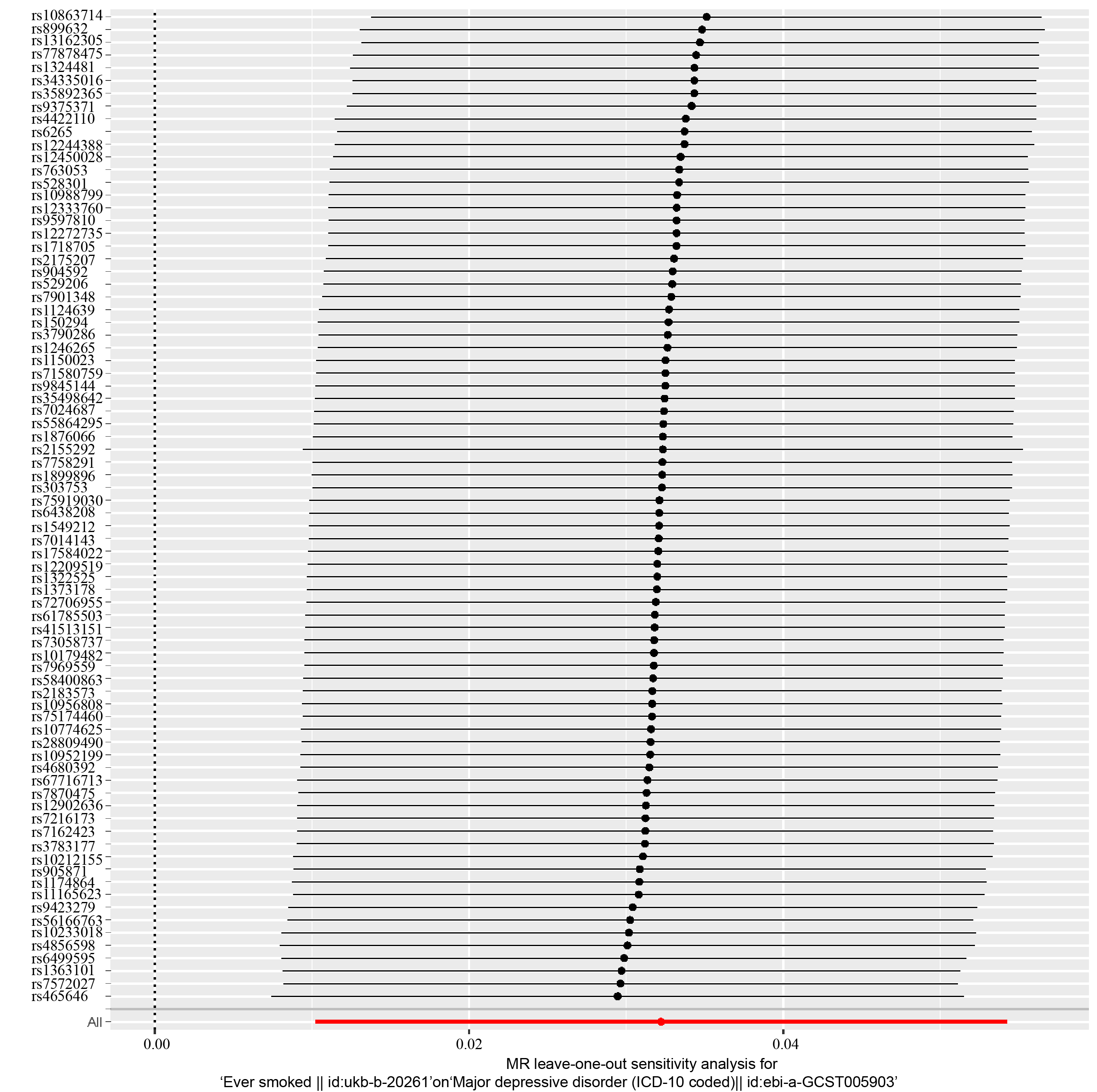

Supplement: Supplementary Figure S1 — Leave-one-out plots of MR analyses for (A) smoking, (B) sleeplessness/insomnia, and (C) BMI on major depressive disease, and for (D) smoking, (E) sleeplessness/insomnia, and (F) BMI on bipolar disorder. [file Image_1.TIF]

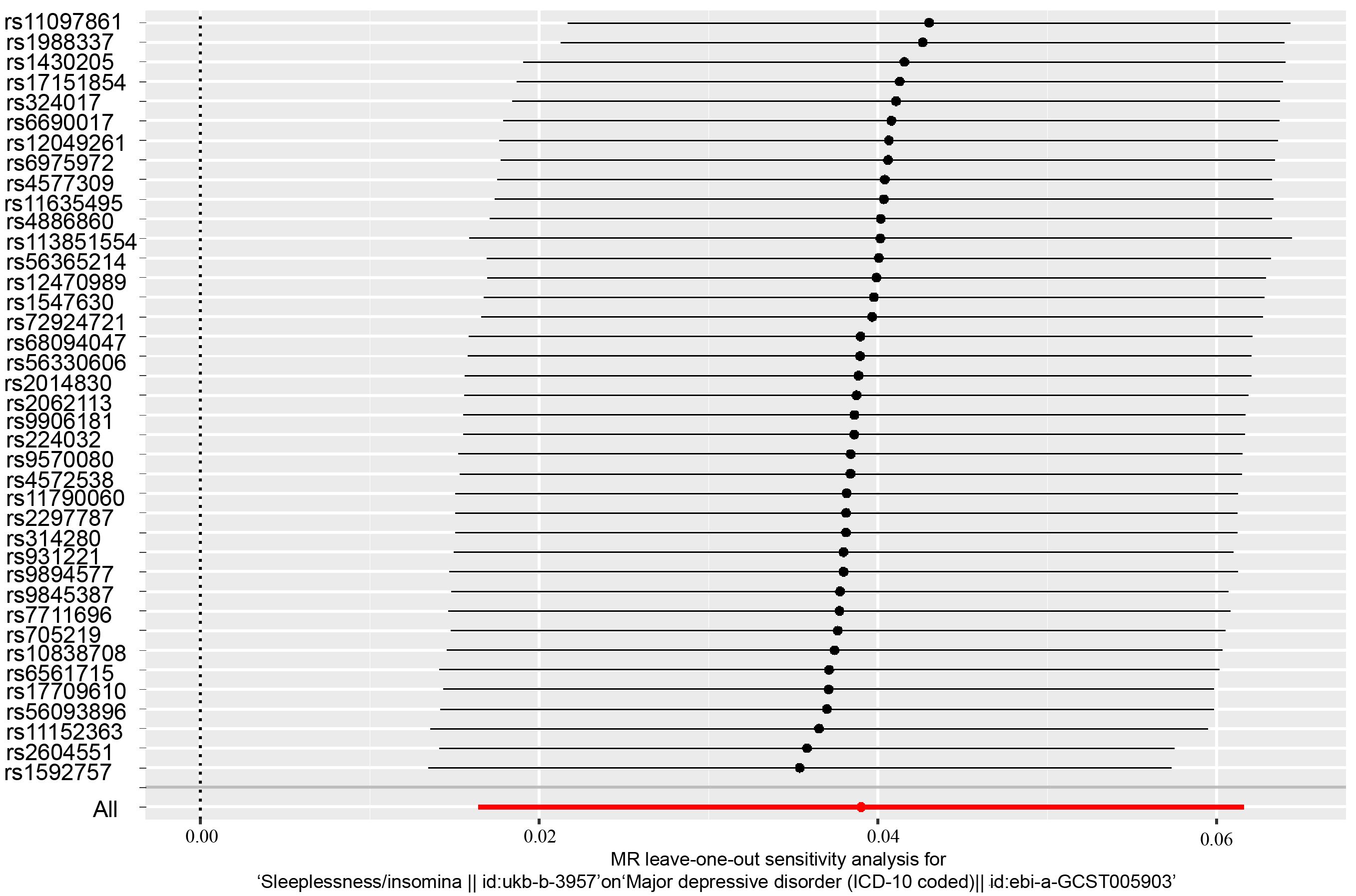

Supplement: Supplementary Figure S2 — Scatter plots of MR analyses for (A) smoking, (B) sleeplessness/insomnia, and (C) BMI on major depressive disease, and for (D) smoking, (E) sleeplessness/insomnia, and (F) BMI on bipolar disorder. [file Image_2.TIF]

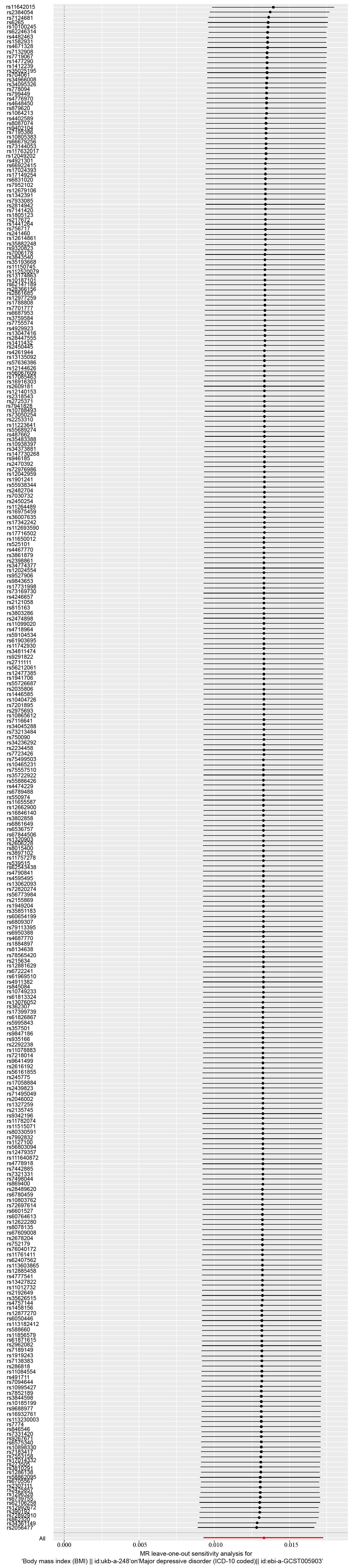

Supplement: Supplementary Figure S3 — Forest plots of MR analyses for (A) smoking, (B) sleeplessness/insomnia, and (C) BMI on major depressive disease, and for (D) smoking, (E) sleeplessness/insomnia, and (F) BMI on bipolar disorder. [file Image_3.TIF]

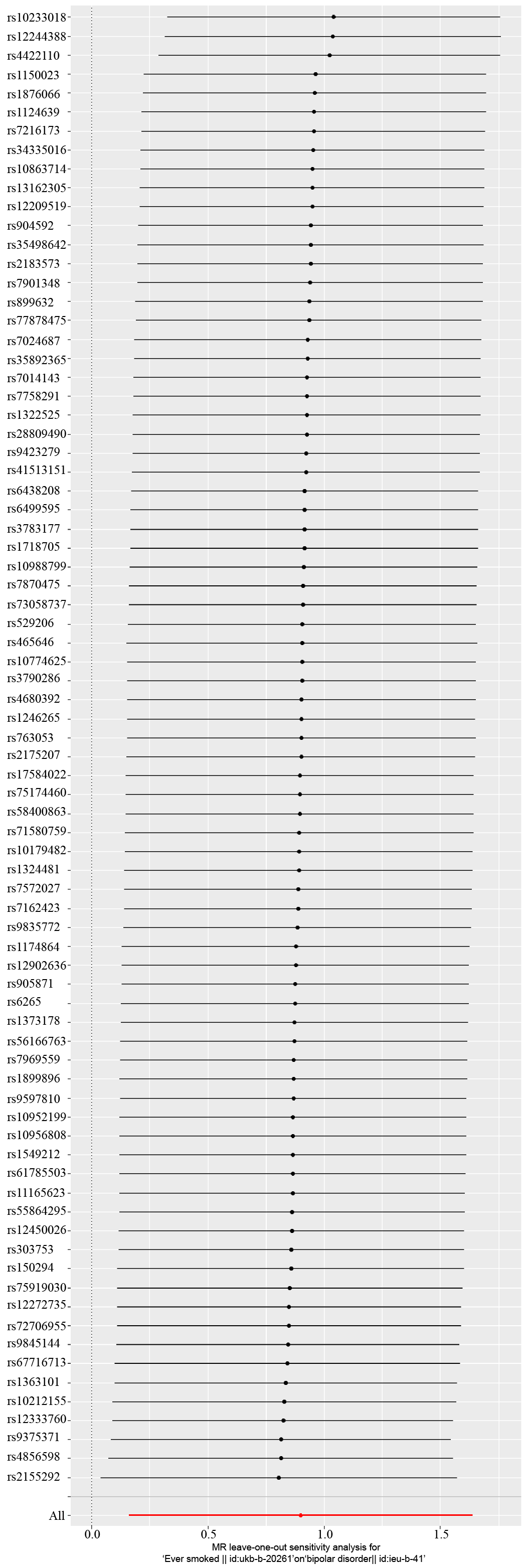

Supplement: Supplementary Figure S4 — Funnel plots of MR analyses for (A) smoking, (B) sleeplessness/insomnia, and (C) BMI on major depressive disease, and for (D) smoking, (E) sleeplessness/insomnia, and (F) BMI on bipolar disorder. [file Image_4.TIF]

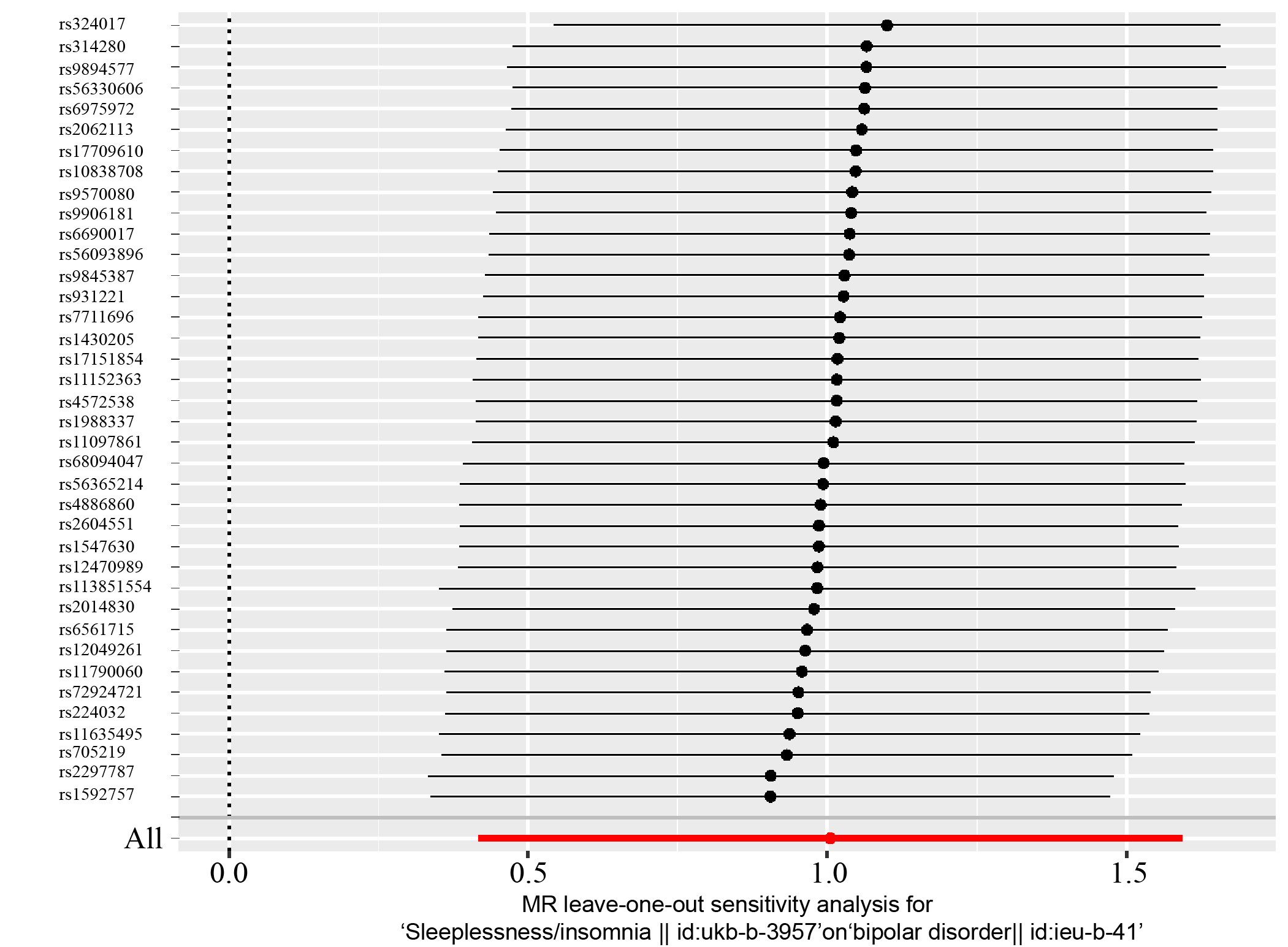

Supplement: Supplementary file 5 [file Image_5.TIF]

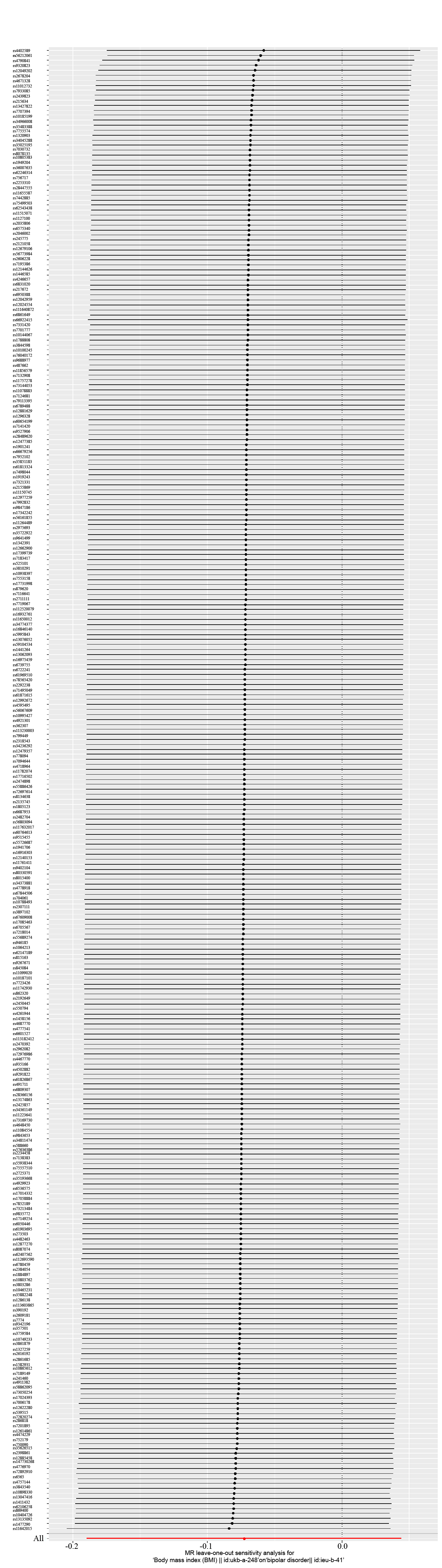

Supplement: Supplementary file 6 [file Image_6.TIF]

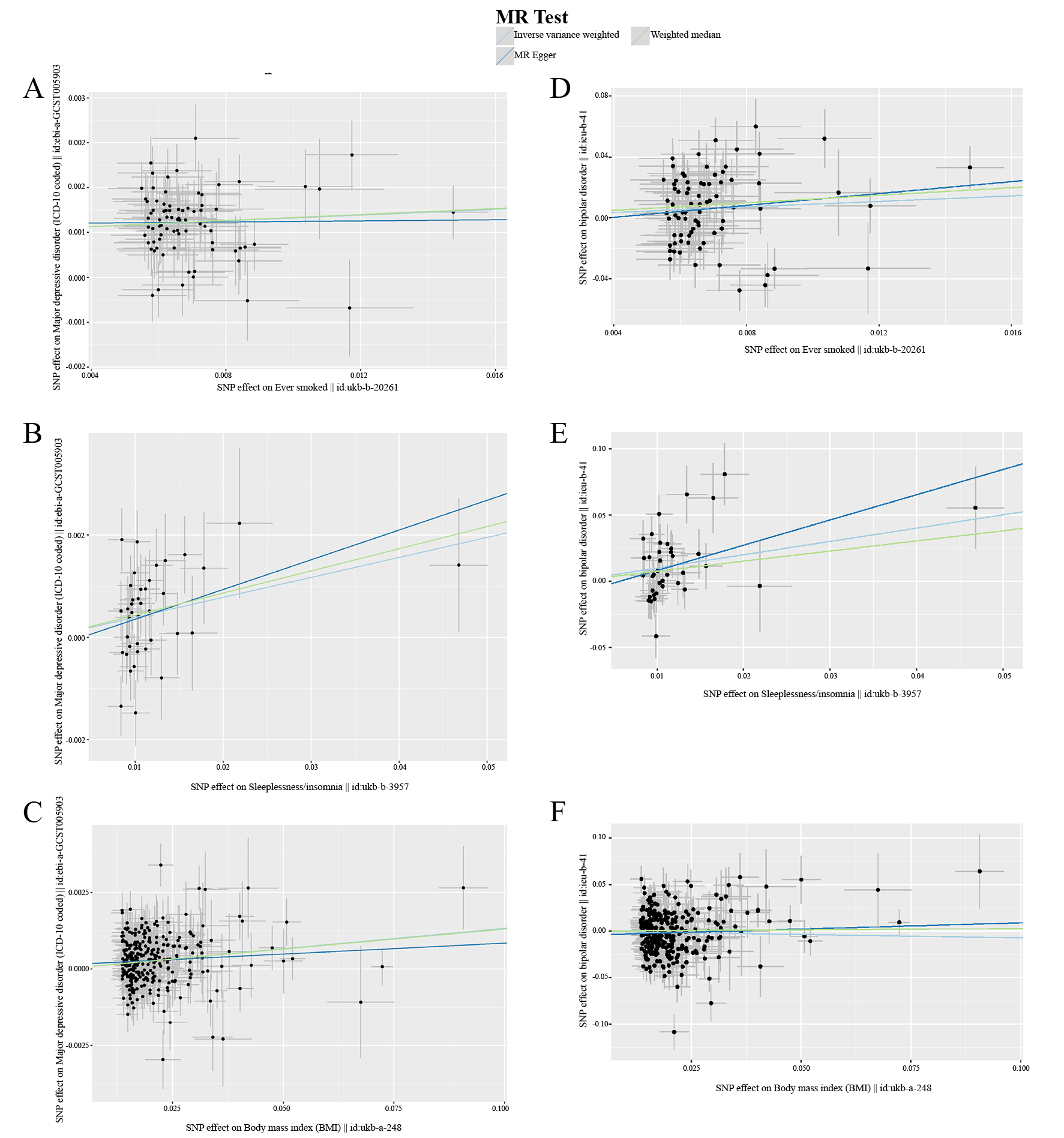

Supplement: Supplementary file 7 [file Image_7.TIF]

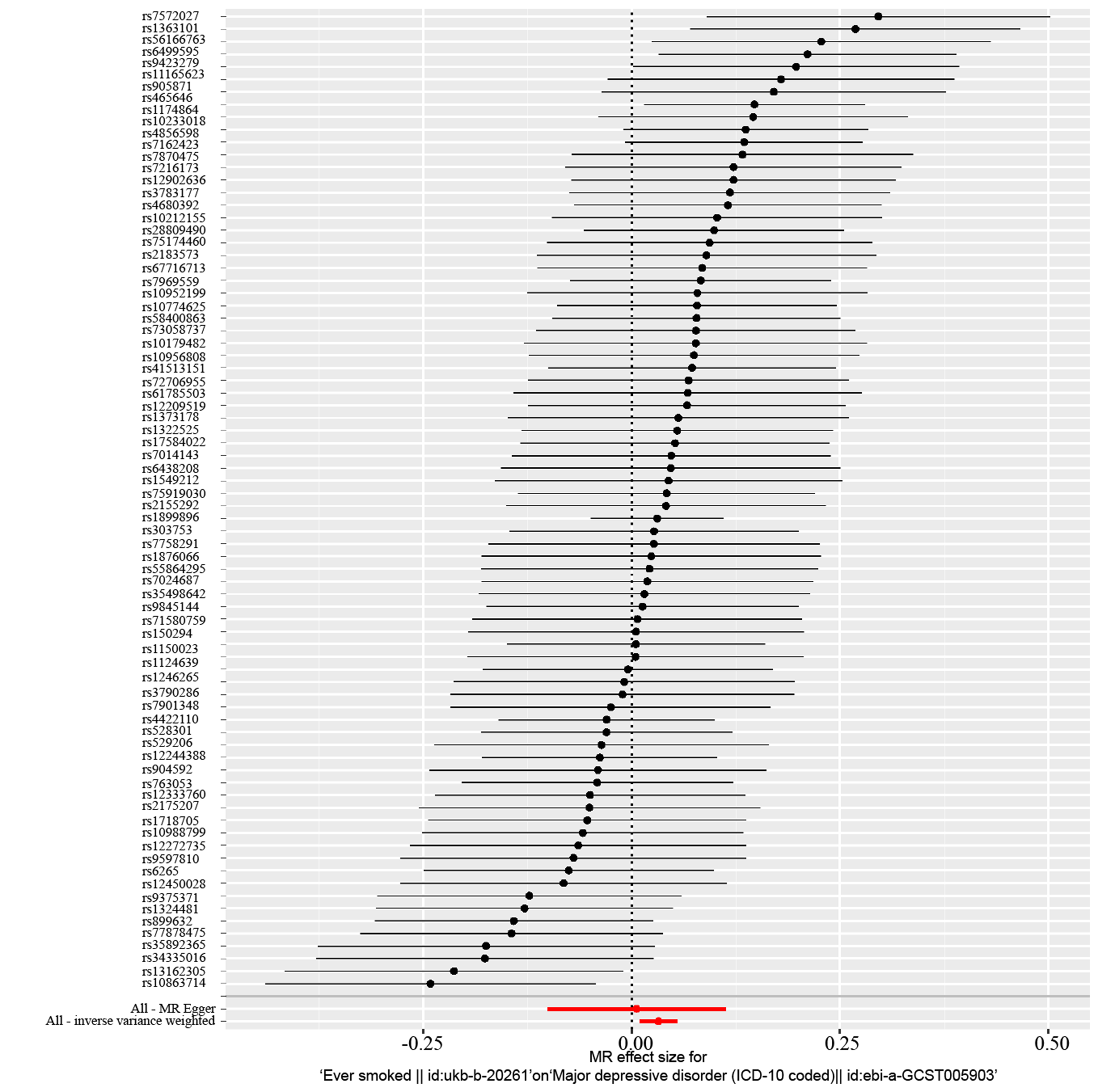

Supplement: Supplementary file 8 [file Image_8.TIF]

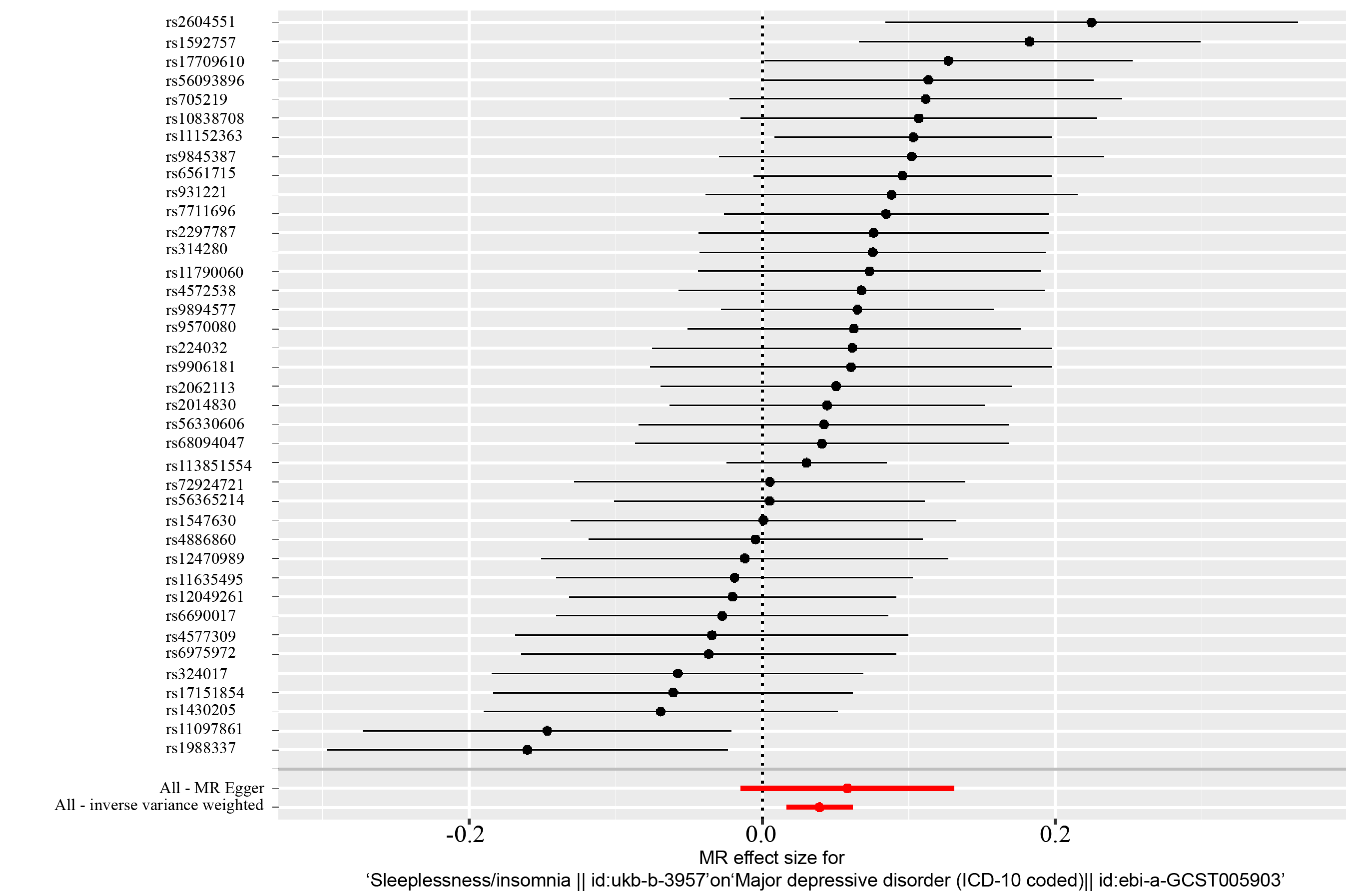

Supplement: Supplementary file 9 [file Image_9.TIF]

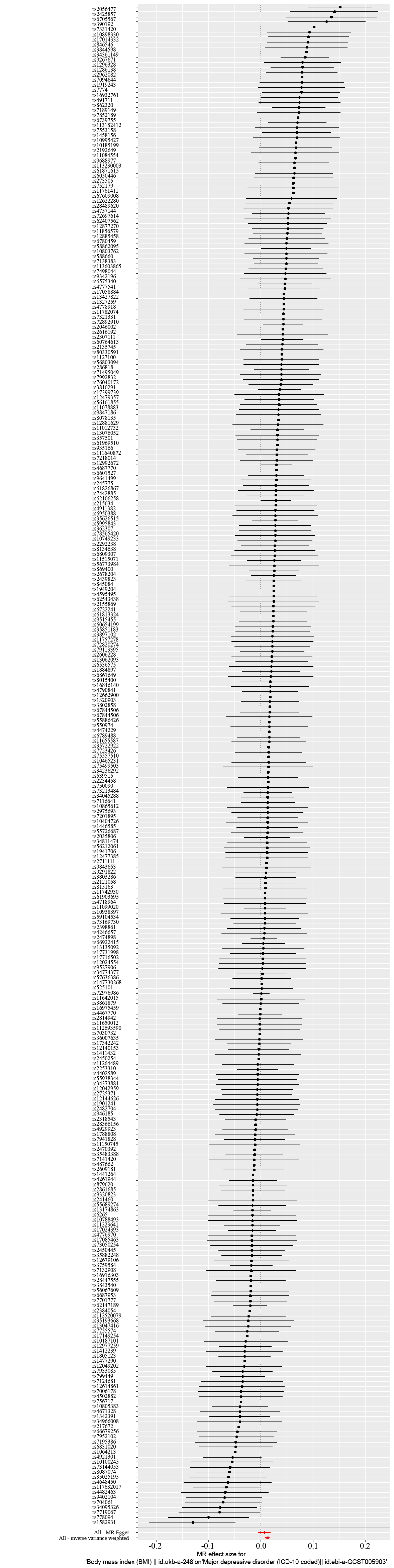

Supplement: Supplementary file 10 [file Image_10.TIF]

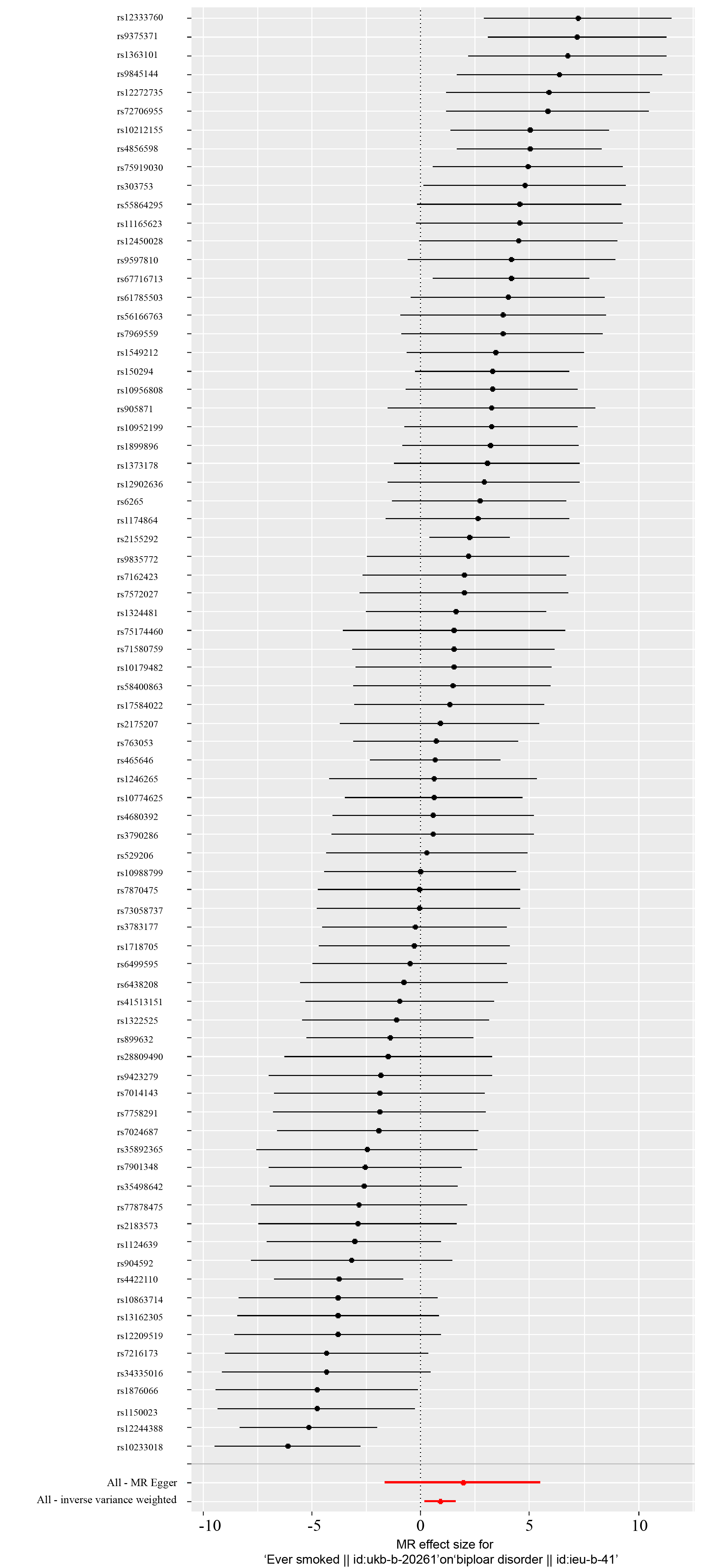

Supplement: Supplementary file 11 [file Image_11.TIF]

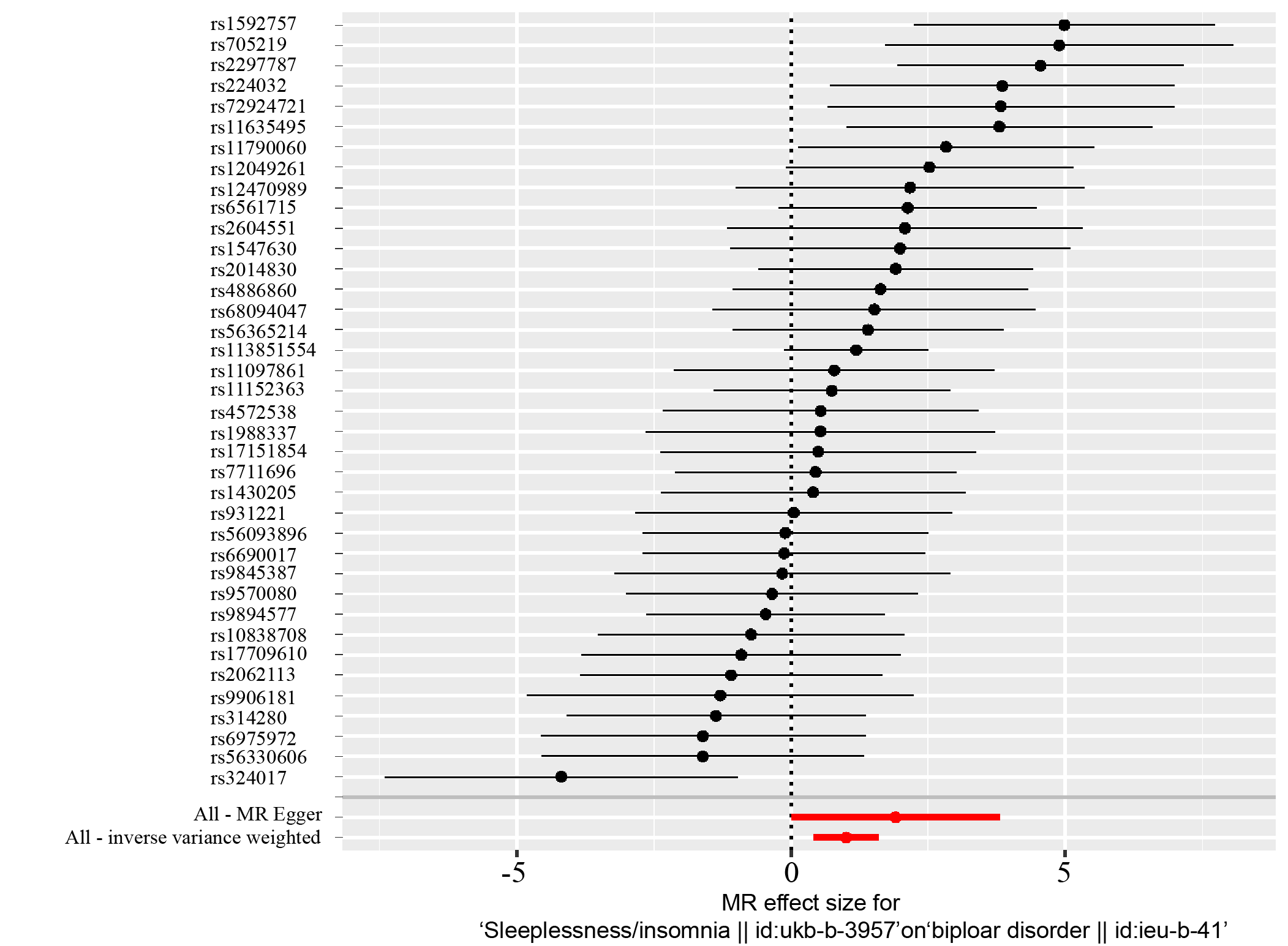

Supplement: Supplementary file 12 [file Image_12.TIF]

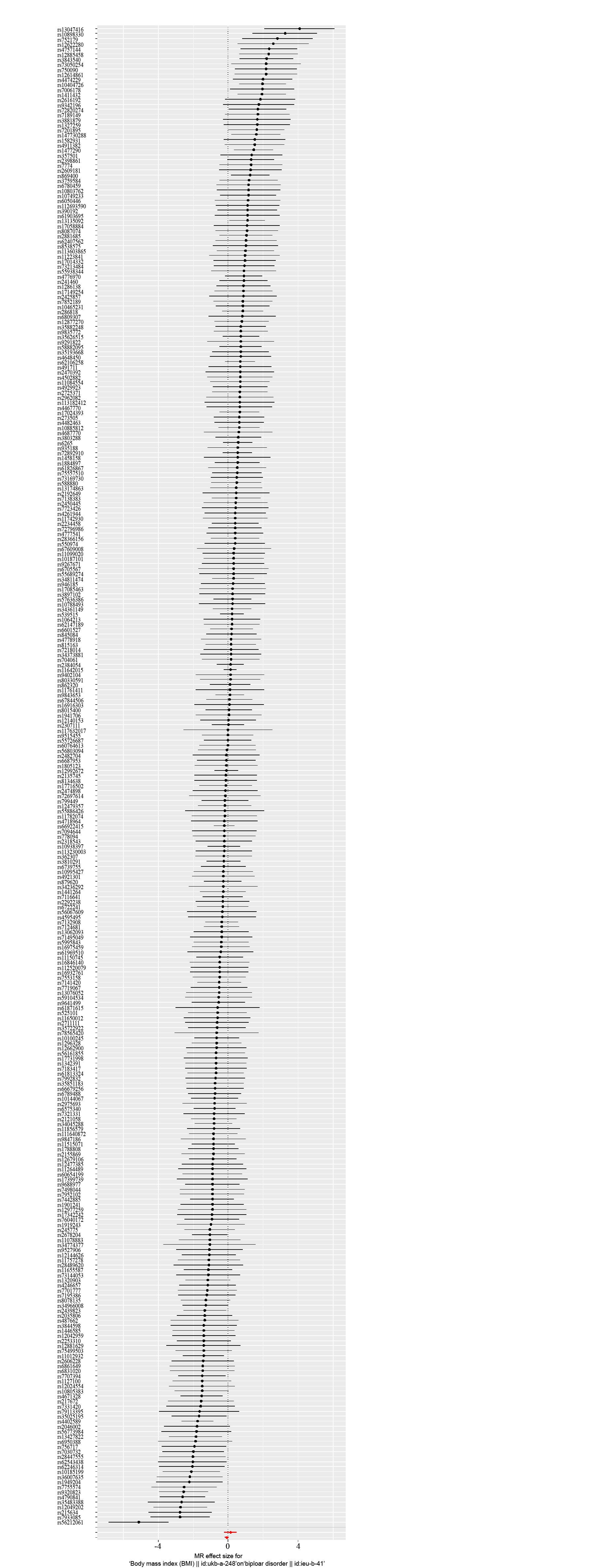

Supplement: Supplementary file 13 [file Image_13.TIF]

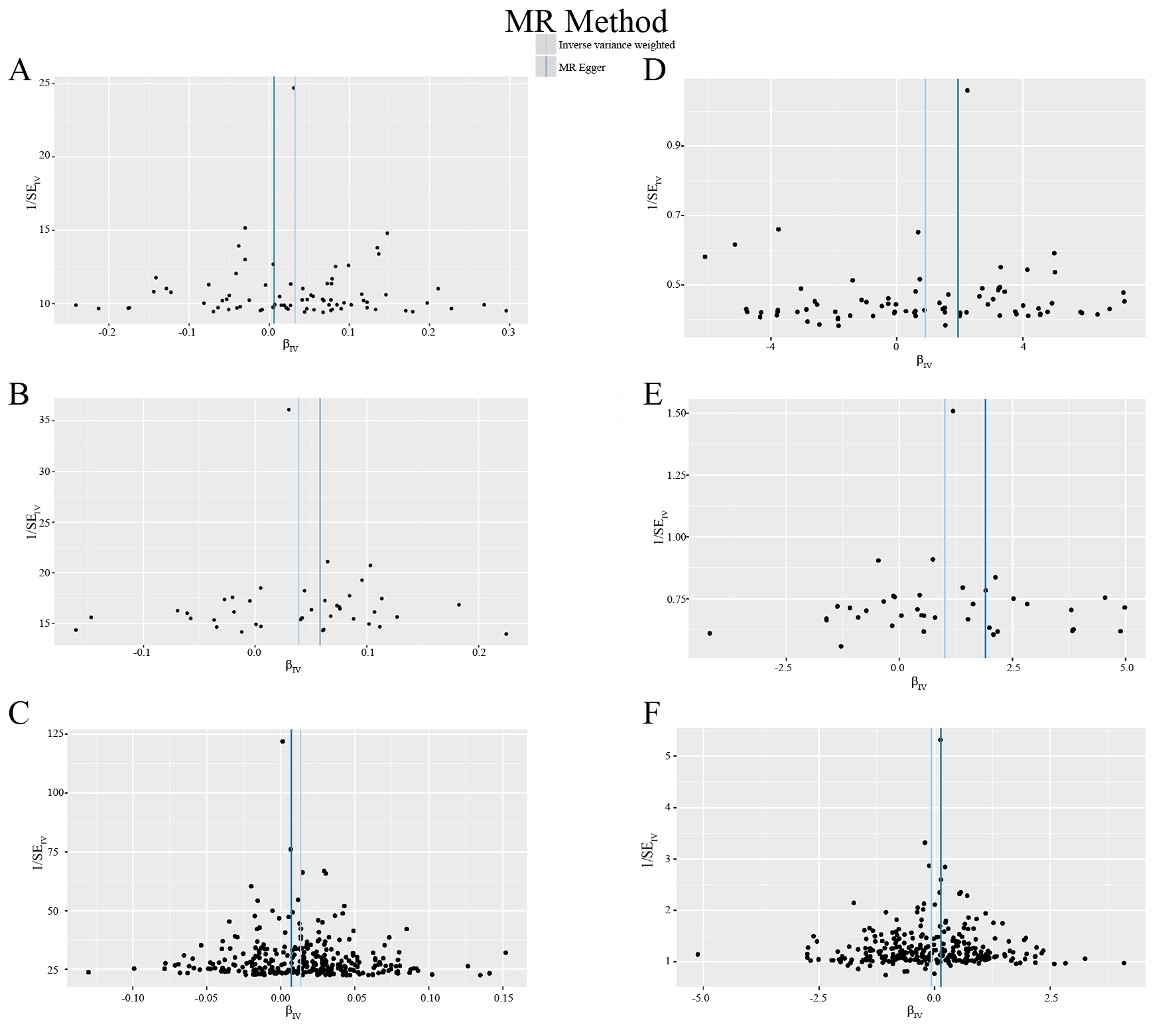

Supplement: Supplementary file 14 [file Image_14.TIF]
