# Supplementary material for: Investigating the causal effects of smoking, sleep, and BMI on major depressive disorder and bipolar disorder: a univariable and multivariable two-sample Mendelian randomization study
Source: Front Psychiatry. 2023 Oct 12;14:1206657. doi: 10.3389/fpsyt.2023.1206657 (PMC10602671; doi:10.3389/fpsyt.2023.1206657)
Supplement: Supplementary file 15 [file Table_1.docx]

Supplementary Tables

Supplementary Table S1. Identification of 78 ever smoke-related SNPs selected as IVs for MDD

| **SNP** | **Chr** | **Position** | **EA** | **OA** | **EAF** | **β** | **SE** | ***p*** | **F** |
| --- | --- | --- | --- | --- | --- | --- | --- | --- | --- |
| rs10179482 | 2 | 81004626 | A | G | 0.485465 | 0.005773 | 0.001007 | 9.90E-09 | 32.8559 |
| rs10212155 | 3 | 117785505 | A | G | 0.852761 | 0.010355 | 0.001423 | 3.40E-13 | 52.97429 |
| rs10233018 | 7 | 117523709 | G | A | 0.503991 | 0.007789 | 0.001005 | 9.20E-15 | 60.06879 |
| rs10774625 | 12 | 111910219 | G | A | 0.503725 | -0.00659 | 0.001003 | 5.00E-11 | 43.15946 |
| rs10863714 | 1 | 208710593 | G | A | 0.576592 | -0.00582 | 0.001016 | 1.00E-08 | 32.77603 |
| rs10952199 | 7 | 1665339 | T | C | 0.426753 | -0.00687 | 0.001017 | 1.40E-11 | 45.68043 |
| rs10956808 | 8 | 92775372 | G | T | 0.422058 | -0.00671 | 0.00102 | 4.70E-11 | 43.31263 |
| rs10988799 | 9 | 102158106 | T | C | 0.528587 | 0.005947 | 0.001008 | 3.60E-09 | 34.82406 |
| rs11165623 | 1 | 96893000 | A | G | 0.504402 | 0.005496 | 0.001004 | 4.40E-08 | 29.96442 |
| rs1124639 | 2 | 200775744 | C | T | 0.554782 | 0.006597 | 0.001011 | 6.70E-11 | 42.61189 |
| rs1150023 | 10 | 9360823 | C | A | 0.737332 | -0.00645 | 0.001143 | 1.60E-08 | 31.88336 |
| rs1174864 | 7 | 53127559 | A | G | 0.550251 | 0.006206 | 0.001011 | 8.40E-10 | 37.66488 |
| rs12209519 | 6 | 67549140 | G | A | 0.408089 | 0.005691 | 0.001026 | 2.90E-08 | 30.75892 |
| rs12244388 | 10 | 104640052 | A | G | 0.338891 | 0.008567 | 0.001061 | 6.80E-16 | 65.18937 |
| rs12272735 | 11 | 7950024 | G | A | 0.392103 | 0.005781 | 0.001027 | 1.80E-08 | 31.66948 |
| rs12333760 | 7 | 99185406 | C | T | 0.165187 | -0.00828 | 0.001355 | 9.90E-10 | 37.34006 |
| rs12450028 | 17 | 2207425 | T | C | 0.34525 | 0.006131 | 0.001055 | 6.30E-09 | 33.73906 |
| rs1246265 | 9 | 86761745 | C | T | 0.69544 | 0.006069 | 0.001094 | 2.90E-08 | 30.79657 |
| rs12902636 | 15 | 47971451 | T | C | 0.502529 | -0.00594 | 0.001008 | 3.70E-09 | 34.77866 |
| rs13162305 | 5 | 12122698 | T | A | 0.335129 | 0.00599 | 0.001071 | 2.20E-08 | 31.28364 |
| rs1322525 | 6 | 19093152 | G | A | 0.36713 | 0.006389 | 0.001043 | 9.10E-10 | 37.50588 |
| rs1324481 | 1 | 33892964 | G | T | 0.681417 | 0.006897 | 0.001079 | 1.60E-10 | 40.87231 |
| rs1363101 | 5 | 103941070 | G | T | 0.520698 | -0.00577 | 0.001005 | 9.30E-09 | 32.97522 |
| rs1373178 | 18 | 49967811 | G | T | 0.594204 | -0.00625 | 0.001027 | 1.20E-09 | 36.96165 |
| rs150294 | 15 | 89931148 | G | A | 0.401317 | -0.00755 | 0.001029 | 2.10E-13 | 53.90795 |
| rs1549212 | 5 | 166996722 | T | C | 0.626251 | -0.00663 | 0.001039 | 1.80E-10 | 40.7145 |
| rs1718705 | 3 | 61823653 | C | G | 0.317331 | 0.006462 | 0.001084 | 2.50E-09 | 35.52037 |
| rs17584022 | 11 | 59190650 | A | G | 0.288018 | -0.00659 | 0.001109 | 2.70E-09 | 35.36412 |
| rs1876066 | 10 | 10040774 | C | T | 0.497781 | -0.00569 | 0.001014 | 1.90E-08 | 31.56592 |
| rs1899896 | 8 | 93201036 | T | C | 0.297037 | 0.007233 | 0.001103 | 5.50E-11 | 42.99655 |
| rs2155292 | 11 | 112910783 | G | A | 0.387722 | 0.014746 | 0.001031 | 2.00E-46 | 204.6931 |
| rs2175207 | 10 | 87357738 | G | A | 0.159486 | 0.007604 | 0.001371 | 2.90E-08 | 30.75037 |
| rs2183573 | 21 | 40574305 | G | A | 0.572269 | -0.00584 | 0.001016 | 9.20E-09 | 32.99727 |
| rs28809490 | 22 | 46414043 | A | G | 0.309077 | -0.00633 | 0.001088 | 5.90E-09 | 33.86212 |
| rs303753 | 18 | 21074922 | A | G | 0.346408 | -0.00608 | 0.001064 | 1.10E-08 | 32.60045 |
| rs34335016 | 1 | 154144681 | T | A | 0.12052 | -0.00864 | 0.001543 | 2.20E-08 | 31.32044 |
| rs35498642 | 3 | 85928634 | T | C | 0.392512 | -0.00624 | 0.001028 | 1.30E-09 | 36.87424 |
| rs35892365 | 8 | 26280685 | T | C | 0.243677 | -0.00671 | 0.001196 | 2.00E-08 | 31.47734 |
| rs3783177 | 13 | 101151360 | G | T | 0.238285 | -0.00729 | 0.001183 | 7.30E-10 | 37.93089 |
| rs3790286 | 20 | 19655938 | C | T | 0.542753 | 0.005591 | 0.001013 | 3.40E-08 | 30.47207 |
| rs41513151 | 7 | 121962809 | A | G | 0.216228 | 0.007256 | 0.001221 | 2.80E-09 | 35.31086 |
| rs4422110 | 2 | 146114898 | T | C | 0.534619 | -0.00884 | 0.001007 | 1.50E-18 | 77.21296 |
| rs465646 | 6 | 111620758 | A | G | 0.84077 | -0.01174 | 0.001374 | 1.30E-17 | 72.96613 |
| rs4680392 | 3 | 157404329 | C | T | 0.673873 | 0.006157 | 0.001073 | 9.60E-09 | 32.91323 |
| rs4856598 | 3 | 85650790 | C | A | 0.62769 | -0.00839 | 0.001049 | 1.20E-15 | 64.06486 |
| rs528301 | 2 | 45154908 | A | G | 0.554847 | 0.007598 | 0.001009 | 5.10E-14 | 56.70959 |
| rs529206 | 11 | 132213460 | T | C | 0.589177 | -0.00585 | 0.001032 | 1.40E-08 | 32.18 |
| rs55864295 | 4 | 70512801 | G | A | 0.185129 | -0.00737 | 0.001291 | 1.10E-08 | 32.62304 |
| rs56166763 | 4 | 140943169 | C | G | 0.368511 | -0.00582 | 0.001041 | 2.30E-08 | 31.2637 |
| rs58400863 | 4 | 31184484 | A | G | 0.341099 | -0.00633 | 0.001064 | 2.70E-09 | 35.35736 |
| rs61785503 | 1 | 50458443 | T | C | 0.23512 | 0.007048 | 0.001183 | 2.50E-09 | 35.52173 |
| rs6265 | 11 | 27679916 | T | C | 0.18847 | -0.00838 | 0.001284 | 6.70E-11 | 42.6048 |
| rs6438208 | 3 | 114170272 | A | G | 0.25141 | -0.00656 | 0.001201 | 4.80E-08 | 29.81511 |
| rs6499595 | 16 | 72934443 | C | G | 0.397268 | -0.00654 | 0.001026 | 1.80E-10 | 40.64059 |
| rs67716713 | 2 | 104267572 | A | C | 0.488327 | -0.00729 | 0.001004 | 4.00E-13 | 52.66509 |
| rs7014143 | 8 | 21587997 | C | A | 0.586417 | 0.006029 | 0.00108 | 2.40E-08 | 31.1518 |
| rs7024687 | 9 | 3174579 | G | A | 0.522154 | -0.00583 | 0.001017 | 1.00E-08 | 32.83601 |
| rs71580759 | 5 | 87770123 | C | T | 0.222323 | -0.00681 | 0.001208 | 1.70E-08 | 31.77684 |
| rs7162423 | 15 | 74052756 | T | C | 0.444196 | -0.00562 | 0.00101 | 2.70E-08 | 30.8948 |
| rs7216173 | 17 | 51891405 | T | A | 0.781979 | -0.00719 | 0.001233 | 5.60E-09 | 33.98522 |
| rs72706955 | 9 | 23818916 | T | C | 0.150042 | -0.00771 | 0.001406 | 4.20E-08 | 30.06384 |
| rs73058737 | 3 | 34532026 | T | G | 0.288033 | 0.006156 | 0.001114 | 3.30E-08 | 30.54651 |
| rs75174460 | 12 | 133486532 | C | T | 0.075101 | 0.010777 | 0.001926 | 2.20E-08 | 31.3032 |
| rs7572027 | 2 | 226286009 | T | C | 0.81255 | 0.007096 | 0.001285 | 3.40E-08 | 30.47902 |
| rs75919030 | 17 | 50193197 | C | T | 0.262188 | -0.0068 | 0.001146 | 2.90E-09 | 35.22657 |
| rs763053 | 16 | 735921 | C | T | 0.225654 | -0.00842 | 0.001206 | 2.90E-12 | 48.72674 |
| rs7758291 | 6 | 129346046 | C | A | 0.684258 | 0.006023 | 0.00108 | 2.40E-08 | 31.1135 |
| rs77878475 | 16 | 18058548 | A | T | 0.084235 | -0.01167 | 0.001875 | 4.90E-10 | 38.72494 |
| rs7870475 | 9 | 128134034 | C | T | 0.475284 | 0.005659 | 0.001005 | 1.80E-08 | 31.68697 |
| rs7901348 | 10 | 63679281 | G | T | 0.551902 | -0.00603 | 0.001017 | 3.10E-09 | 35.12575 |
| rs7969559 | 12 | 69655167 | G | A | 0.720249 | -0.00622 | 0.001118 | 2.60E-08 | 30.97396 |
| rs899632 | 4 | 57749347 | C | T | 0.389049 | -0.00703 | 0.001033 | 9.90E-12 | 46.33967 |
| rs904592 | 3 | 25193102 | T | C | 0.482639 | 0.005692 | 0.001013 | 1.90E-08 | 31.56026 |
| rs905871 | 11 | 4661285 | G | A | 0.339517 | -0.00584 | 0.001061 | 3.70E-08 | 30.28003 |
| rs9375371 | 6 | 98751680 | A | G | 0.269366 | 0.007068 | 0.001134 | 4.60E-10 | 38.82325 |
| rs9423279 | 10 | 125680419 | G | C | 0.656563 | -0.00627 | 0.001078 | 5.90E-09 | 33.88344 |
| rs9597810 | 13 | 59264524 | G | C | 0.327261 | -0.00587 | 0.001072 | 4.30E-08 | 30.01478 |
| rs9845144 | 3 | 184056716 | A | G | 0.272671 | 0.006558 | 0.00114 | 8.80E-09 | 33.088 |

Supplementary Table S2. Identification of 39 sleeplessness/insomnia-related SNPs selected as IVs for MDD

| **SNP** | **Chr** | **Position** | **EA** | **OA** | **EAF** | **β** | **SE** | ***p*** | **F** |
| --- | --- | --- | --- | --- | --- | --- | --- | --- | --- |
| rs10838708 | 11 | 47441513 | A | G | 0.45899 | -0.00948 | 0.001503 | 2.90E-10 | 39.76959 |
| rs11097861 | 4 | 105330133 | G | A | 0.716256 | 0.010044 | 0.001649 | 1.10E-09 | 37.07694 |
| rs11152363 | 18 | 53057188 | A | G | 0.186319 | 0.015639 | 0.001925 | 4.50E-16 | 65.9921 |
| rs113851554 | 2 | 66750564 | T | G | 0.057291 | 0.04678 | 0.003313 | 2.90E-45 | 199.345 |
| rs11635495 | 15 | 67804682 | C | T | 0.512179 | 0.009373 | 0.001485 | 2.80E-10 | 39.83737 |
| rs11790060 | 9 | 96202932 | C | T | 0.330834 | -0.01034 | 0.001579 | 5.80E-11 | 42.8997 |
| rs12049261 | 1 | 107190407 | C | G | 0.292534 | 0.011187 | 0.00163 | 6.80E-12 | 47.07397 |
| rs12470989 | 2 | 200955823 | G | A | 0.203934 | -0.01024 | 0.001845 | 2.80E-08 | 30.82647 |
| rs1430205 | 5 | 87678585 | T | C | 0.461507 | 0.009475 | 0.001491 | 2.10E-10 | 40.37904 |
| rs1547630 | 13 | 112642528 | A | G | 0.651513 | 0.009108 | 0.001564 | 5.80E-09 | 33.89416 |
| rs1592757 | 5 | 103889998 | C | G | 0.355788 | 0.010222 | 0.00155 | 4.30E-11 | 43.49074 |
| rs17151854 | 8 | 10236559 | T | G | 0.15241 | 0.012989 | 0.002074 | 3.80E-10 | 39.21133 |
| rs17709610 | 10 | 104250278 | G | A | 0.297979 | -0.00992 | 0.001621 | 9.50E-10 | 37.43119 |
| rs1988337 | 4 | 91292200 | G | A | 0.552395 | 0.008387 | 0.001496 | 2.10E-08 | 31.41346 |
| rs2014830 | 3 | 50172397 | T | C | 0.303519 | -0.0116 | 0.001623 | 8.90E-13 | 51.08027 |
| rs2062113 | 16 | 59476179 | C | T | 0.568257 | -0.00962 | 0.001503 | 1.60E-10 | 40.95302 |
| rs224032 | 10 | 64521829 | A | G | 0.550358 | 0.008391 | 0.001491 | 1.80E-08 | 31.66579 |
| rs2297787 | 10 | 104680137 | A | T | 0.080193 | -0.0178 | 0.00275 | 9.60E-11 | 41.89789 |
| rs2604551 | 4 | 15091201 | G | T | 0.640384 | -0.00848 | 0.001552 | 4.70E-08 | 29.84569 |
| rs314280 | 6 | 105400837 | G | A | 0.547047 | 0.009714 | 0.001491 | 7.30E-11 | 42.42382 |
| rs324017 | 12 | 57487814 | C | A | 0.705433 | -0.00988 | 0.001631 | 1.40E-09 | 36.6931 |
| rs4572538 | 2 | 147423012 | T | C | 0.364055 | -0.00961 | 0.001562 | 7.70E-10 | 37.83872 |
| rs4577309 | 2 | 191288833 | G | A | 0.533655 | -0.00855 | 0.001492 | 1.00E-08 | 32.82037 |
| rs4886860 | 15 | 74340336 | C | G | 0.767408 | -0.0118 | 0.001756 | 1.80E-11 | 45.14282 |
| rs56093896 | 2 | 114103966 | A | C | 0.214053 | -0.01241 | 0.001814 | 7.70E-12 | 46.83557 |
| rs56330606 | 19 | 37673953 | G | A | 0.378954 | 0.009309 | 0.00153 | 1.20E-09 | 37.00013 |
| rs56365214 | 2 | 58930167 | A | C | 0.155781 | -0.01479 | 0.002052 | 5.60E-13 | 51.97059 |
| rs6561715 | 13 | 53888526 | A | T | 0.630662 | -0.01162 | 0.001542 | 4.80E-14 | 56.79057 |
| rs6690017 | 1 | 57842729 | G | T | 0.408855 | -0.01027 | 0.00151 | 1.10E-11 | 46.21663 |
| rs68094047 | 12 | 109855201 | T | C | 0.251267 | 0.010336 | 0.001717 | 1.70E-09 | 36.24788 |
| rs6975972 | 7 | 1070468 | G | A | 0.578726 | -0.00902 | 0.001504 | 2.00E-09 | 35.95997 |
| rs705219 | 3 | 117702270 | A | T | 0.887373 | 0.013423 | 0.002353 | 1.20E-08 | 32.54311 |
| rs72924721 | 11 | 65585990 | T | C | 0.073137 | 0.016478 | 0.002881 | 1.10E-08 | 32.71248 |
| rs7711696 | 5 | 135486536 | T | G | 0.305042 | 0.011172 | 0.001611 | 4.10E-12 | 48.06565 |
| rs931221 | 12 | 84721107 | A | T | 0.236738 | 0.010636 | 0.001753 | 1.30E-09 | 36.80752 |
| rs9570080 | 13 | 59832775 | C | T | 0.344131 | -0.01064 | 0.001579 | 1.60E-11 | 45.41284 |
| rs9845387 | 3 | 116425935 | A | C | 0.040279 | -0.02186 | 0.003776 | 7.10E-09 | 33.49461 |
| rs9894577 | 17 | 43223292 | A | G | 0.3182 | 0.013205 | 0.001597 | 1.30E-16 | 68.38239 |
| rs9906181 | 17 | 21297686 | G | A | 0.687567 | -0.00915 | 0.001639 | 2.40E-08 | 31.1798 |

Supplementary Table S3. Identification of 302 BMI-related SNPs selected as IVs for MDD

| **SNP** | **Chr** | **Position** | **EA** | **OA** | **EAF** | **β** | **SE** | ***p*** | **F** |
| --- | --- | --- | --- | --- | --- | --- | --- | --- | --- |
| rs10100245 | 8 | 77226919 | A | G | 0.56644 | 0.020637 | 0.002423 | 1.64E-17 | 72.53998 |
| rs10185199 | 2 | 40282202 | A | G | 0.27894 | -0.01871 | 0.002749 | 1.01E-11 | 46.3119 |
| rs10187101 | 2 | 50742227 | T | C | 0.359822 | -0.01571 | 0.002505 | 3.54E-10 | 39.3555 |
| rs10404726 | 19 | 18834514 | T | C | 0.466784 | -0.01986 | 0.002407 | 1.60E-16 | 68.05188 |
| rs10465231 | 9 | 92183413 | T | C | 0.5494 | 0.01714 | 0.002427 | 1.65E-12 | 49.86364 |
| rs1064213 | 2 | 198950240 | A | G | 0.476968 | 0.016189 | 0.002406 | 1.70E-11 | 45.28867 |
| rs10749233 | 10 | 118777998 | C | G | 0.75947 | -0.01896 | 0.002848 | 2.75E-11 | 44.34695 |
| rs10788493 | 10 | 88104837 | T | C | 0.45676 | 0.013692 | 0.00242 | 1.53E-08 | 32.01386 |
| rs10803762 | 2 | 161105876 | A | G | 0.678865 | 0.015464 | 0.002582 | 2.11E-09 | 35.87415 |
| rs10805383 | 5 | 63034606 | A | G | 0.479558 | 0.016949 | 0.002409 | 1.99E-12 | 49.50236 |
| rs10865612 | 3 | 85868529 | C | T | 0.354736 | -0.02308 | 0.002511 | 3.80E-20 | 84.53157 |
| rs10898330 | 11 | 84630335 | T | C | 0.525392 | -0.01429 | 0.002417 | 3.39E-09 | 34.94931 |
| rs10938397 | 4 | 45182527 | G | A | 0.434075 | 0.028953 | 0.002429 | 9.55E-33 | 142.0675 |
| rs10995427 | 10 | 64842838 | A | G | 0.360827 | -0.01653 | 0.002516 | 5.00E-11 | 43.18012 |
| rs11012732 | 10 | 21830104 | G | A | 0.331106 | 0.02385 | 0.002555 | 1.03E-20 | 87.12295 |
| rs11078883 | 17 | 2138828 | G | C | 0.34744 | 0.015679 | 0.002532 | 5.96E-10 | 38.33534 |
| rs11084554 | 19 | 31019807 | A | G | 0.156586 | -0.02158 | 0.003304 | 6.58E-11 | 42.64196 |
| rs11099020 | 4 | 130724902 | T | C | 0.642739 | -0.01532 | 0.002512 | 1.06E-09 | 37.2054 |
| rs11150745 | 17 | 78757626 | G | A | 0.319489 | -0.02098 | 0.002579 | 4.13E-16 | 66.17944 |
| rs111640872 | 19 | 30290357 | C | G | 0.331076 | 0.020623 | 0.002559 | 7.72E-16 | 64.94699 |
| rs11223641 | 11 | 133820022 | C | T | 0.144615 | -0.0191 | 0.003432 | 2.61E-08 | 30.98062 |
| rs112520079 | 8 | 95594425 | G | T | 0.198951 | 0.020458 | 0.003014 | 1.14E-11 | 46.06922 |
| rs11264489 | 1 | 156480831 | G | A | 0.361994 | 0.014085 | 0.002507 | 1.93E-08 | 31.56273 |
| rs112693590 | 19 | 46274553 | A | G | 0.049707 | -0.03292 | 0.00568 | 6.80E-09 | 33.59166 |
| rs1127100 | 1 | 32193647 | C | T | 0.648239 | 0.016492 | 0.002522 | 6.20E-11 | 42.76012 |
| rs113182412 | 15 | 66503584 | A | G | 0.165919 | -0.01937 | 0.003284 | 3.68E-09 | 34.78633 |
| rs113230003 | 19 | 18460956 | A | G | 0.260185 | -0.01978 | 0.002759 | 7.56E-13 | 51.39759 |
| rs113603865 | 1 | 39564930 | T | C | 0.212428 | 0.01975 | 0.002957 | 2.42E-11 | 44.60028 |
| rs11515071 | 9 | 15855545 | T | C | 0.365135 | -0.02281 | 0.002506 | 8.84E-20 | 82.86212 |
| rs11642015 | 16 | 53802494 | T | C | 0.40236 | 0.072369 | 0.002447 | 6.40E-192 | 874.3937 |
| rs11650012 | 17 | 52927879 | A | T | 0.169618 | 0.018609 | 0.003218 | 7.37E-09 | 33.43519 |
| rs11655587 | 17 | 47140794 | T | C | 0.359868 | -0.01964 | 0.002511 | 5.26E-15 | 61.16698 |
| rs11742930 | 5 | 105774098 | T | C | 0.566428 | 0.014308 | 0.002429 | 3.84E-09 | 34.70479 |
| rs11757278 | 6 | 13180454 | C | T | 0.305848 | -0.01579 | 0.002609 | 1.42E-09 | 36.64304 |
| rs11761411 | 7 | 39303649 | T | C | 0.154361 | -0.01848 | 0.00334 | 3.17E-08 | 30.60168 |
| rs117632017 | 15 | 52260107 | A | G | 0.039142 | 0.036382 | 0.006447 | 1.67E-08 | 31.84932 |
| rs11782074 | 8 | 142617096 | T | G | 0.385641 | 0.014495 | 0.002507 | 7.37E-09 | 33.43542 |
| rs11856579 | 15 | 78012688 | A | G | 0.268192 | -0.01979 | 0.002711 | 2.91E-13 | 53.27422 |
| rs12024554 | 1 | 19925759 | T | C | 0.236905 | -0.01627 | 0.002827 | 8.75E-09 | 33.10169 |
| rs12042959 | 1 | 243533273 | G | A | 0.145777 | -0.02056 | 0.003425 | 1.96E-09 | 36.01878 |
| rs12049202 | 1 | 77967523 | T | C | 0.198651 | 0.021836 | 0.003008 | 3.91E-13 | 52.69047 |
| rs12140153 | 1 | 62579891 | T | G | 0.096761 | -0.03182 | 0.004168 | 2.25E-14 | 58.30603 |
| rs12144626 | 1 | 47670525 | C | T | 0.581821 | -0.01721 | 0.002444 | 1.87E-12 | 49.61626 |
| rs12477385 | 2 | 166144850 | T | G | 0.2273 | -0.01725 | 0.002876 | 1.99E-09 | 35.98089 |
| rs12479357 | 2 | 181570507 | G | A | 0.631081 | 0.017865 | 0.002499 | 8.71E-13 | 51.11822 |
| rs12614861 | 2 | 175047184 | T | G | 0.34701 | 0.015234 | 0.002522 | 1.53E-09 | 36.49208 |
| rs12622280 | 2 | 79515954 | G | T | 0.157075 | -0.0187 | 0.003305 | 1.53E-08 | 32.01071 |
| rs12662900 | 6 | 97757782 | A | T | 0.282225 | -0.01686 | 0.002673 | 2.85E-10 | 39.77773 |
| rs12679106 | 8 | 73443198 | T | G | 0.711341 | -0.02203 | 0.002665 | 1.38E-16 | 68.33847 |
| rs1286138 | 14 | 91485445 | G | T | 0.673439 | 0.018051 | 0.002564 | 1.92E-12 | 49.56318 |
| rs12877270 | 13 | 97047020 | A | G | 0.43845 | 0.016908 | 0.002439 | 4.15E-12 | 48.05751 |
| rs12881629 | 14 | 101146413 | G | A | 0.082146 | 0.023968 | 0.004366 | 4.04E-08 | 30.13188 |
| rs12885458 | 14 | 47303114 | G | T | 0.509982 | -0.01561 | 0.002404 | 8.40E-11 | 42.16455 |
| rs1296328 | 4 | 137083193 | C | A | 0.559914 | -0.01898 | 0.002431 | 5.86E-15 | 60.95502 |
| rs12977259 | 19 | 1951123 | G | A | 0.821338 | 0.018626 | 0.003161 | 3.80E-09 | 34.7264 |
| rs12992672 | 2 | 632592 | A | G | 0.828635 | 0.050781 | 0.003182 | 2.70E-57 | 254.6103 |
| rs13047416 | 21 | 40309436 | G | C | 0.375427 | -0.01364 | 0.002489 | 4.24E-08 | 30.03788 |
| rs13062093 | 3 | 35667057 | G | T | 0.366444 | 0.01706 | 0.002491 | 7.42E-12 | 46.91575 |
| rs13076052 | 3 | 44456573 | G | C | 0.277037 | 0.016077 | 0.002701 | 2.65E-09 | 35.4261 |
| rs13135092 | 4 | 103198082 | G | A | 0.083234 | 0.050055 | 0.004374 | 2.60E-30 | 130.9321 |
| rs13174863 | 5 | 139080745 | G | A | 0.148208 | 0.024979 | 0.003401 | 2.08E-13 | 53.93468 |
| rs1320903 | 3 | 131758077 | A | G | 0.318441 | 0.022008 | 0.002581 | 1.52E-17 | 72.69441 |
| rs1327259 | 6 | 51177811 | G | A | 0.38613 | -0.014 | 0.002475 | 1.54E-08 | 32.00164 |
| rs1342391 | 1 | 96285385 | T | G | 0.669963 | 0.015869 | 0.002561 | 5.83E-10 | 38.37963 |
| rs13427822 | 2 | 213414265 | G | A | 0.272052 | -0.0199 | 0.002729 | 3.05E-13 | 53.18003 |
| rs1411432 | 9 | 16728532 | C | A | 0.183895 | 0.024946 | 0.003109 | 1.02E-15 | 64.39497 |
| rs1412239 | 9 | 28425515 | G | C | 0.324541 | 0.023921 | 0.002563 | 1.02E-20 | 87.12825 |
| rs1441264 | 13 | 79580919 | A | G | 0.591866 | 0.020638 | 0.002497 | 1.39E-16 | 68.33101 |
| rs1446585 | 2 | 136407479 | G | A | 0.228006 | -0.01711 | 0.002877 | 2.71E-09 | 35.38576 |
| rs1458156 | 12 | 41887940 | T | C | 0.488264 | 0.013785 | 0.002405 | 9.99E-09 | 32.84591 |
| rs1477290 | 5 | 87988934 | C | T | 0.135362 | 0.033539 | 0.003537 | 2.51E-21 | 89.9115 |
| rs147730268 | 12 | 123024476 | T | G | 0.09053 | -0.03611 | 0.004329 | 7.38E-17 | 69.57578 |
| rs1582931 | 5 | 122657199 | A | G | 0.472057 | -0.01486 | 0.002426 | 8.97E-10 | 37.53852 |
| rs16846140 | 2 | 212292521 | G | A | 0.33821 | 0.01619 | 0.002544 | 1.98E-10 | 40.49305 |
| rs16916303 | 9 | 30823761 | G | A | 0.119234 | -0.02072 | 0.003739 | 3.01E-08 | 30.70542 |
| rs16932761 | 8 | 67202787 | A | G | 0.251546 | -0.01797 | 0.002782 | 1.04E-10 | 41.75247 |
| rs16975459 | 18 | 39548277 | C | A | 0.121305 | 0.023972 | 0.003686 | 7.84E-11 | 42.30012 |
| rs17014332 | 1 | 209520450 | C | T | 0.210609 | 0.018029 | 0.002944 | 9.16E-10 | 37.49863 |
| rs17024393 | 1 | 110154688 | C | T | 0.025799 | 0.067508 | 0.007598 | 6.45E-19 | 78.93554 |
| rs17058884 | 9 | 71527774 | G | T | 0.045005 | -0.03183 | 0.005808 | 4.27E-08 | 30.02205 |
| rs17085463 | 4 | 65740387 | A | G | 0.317007 | -0.01481 | 0.002598 | 1.19E-08 | 32.50863 |
| rs17149254 | 7 | 76634463 | C | T | 0.808564 | -0.02381 | 0.003116 | 2.17E-14 | 58.37463 |
| rs17342242 | 8 | 60828697 | G | A | 0.229795 | -0.01616 | 0.002866 | 1.71E-08 | 31.8055 |
| rs17399739 | 10 | 87490850 | G | A | 0.069526 | 0.026237 | 0.004722 | 2.76E-08 | 30.86799 |
| rs17716502 | 8 | 116659731 | T | C | 0.207243 | -0.0223 | 0.002986 | 8.16E-14 | 55.77204 |
| rs17731998 | 3 | 82669493 | T | C | 0.269878 | 0.016159 | 0.002703 | 2.26E-09 | 35.73554 |
| rs1788808 | 18 | 21090023 | G | A | 0.497341 | -0.01831 | 0.002405 | 2.67E-14 | 57.9653 |
| rs1805123 | 7 | 150645534 | G | T | 0.246217 | -0.01799 | 0.002788 | 1.11E-10 | 41.6287 |
| rs1884897 | 20 | 6612832 | G | A | 0.628254 | 0.021329 | 0.002492 | 1.15E-17 | 73.23943 |
| rs1901241 | 12 | 97941390 | G | A | 0.160137 | 0.019292 | 0.00329 | 4.54E-09 | 34.38017 |
| rs1919243 | 5 | 88778861 | C | T | 0.487067 | 0.013873 | 0.002433 | 1.19E-08 | 32.50127 |
| rs1941706 | 18 | 31223776 | G | A | 0.463511 | 0.013941 | 0.002411 | 7.36E-09 | 33.43948 |
| rs1949204 | 13 | 65475834 | G | T | 0.761765 | 0.016653 | 0.002819 | 3.50E-09 | 34.8872 |
| rs2035806 | 10 | 133984916 | A | G | 0.566354 | -0.01663 | 0.002428 | 7.47E-12 | 46.9039 |
| rs2046002 | 15 | 95272235 | C | T | 0.639044 | -0.01457 | 0.002508 | 6.24E-09 | 33.76007 |
| rs2056477 | 7 | 2079744 | C | G | 0.22589 | -0.02232 | 0.002876 | 8.56E-15 | 60.20826 |
| rs2121058 | 13 | 58627256 | C | T | 0.228644 | -0.02381 | 0.002861 | 8.49E-17 | 69.29907 |
| rs2135745 | 9 | 109150784 | G | C | 0.753229 | -0.01684 | 0.002801 | 1.85E-09 | 36.13052 |
| rs2155869 | 18 | 13199302 | C | T | 0.819163 | -0.01845 | 0.003111 | 3.06E-09 | 35.14722 |
| rs215634 | 7 | 32369148 | G | A | 0.613873 | -0.01477 | 0.002476 | 2.45E-09 | 35.57906 |
| rs217672 | 14 | 62361021 | C | A | 0.272013 | 0.015866 | 0.002706 | 4.52E-09 | 34.38773 |
| rs2192649 | 7 | 78126279 | G | T | 0.497183 | 0.013669 | 0.002416 | 1.54E-08 | 32.00719 |
| rs2234458 | 11 | 65639374 | T | C | 0.639151 | -0.02064 | 0.002496 | 1.35E-16 | 68.3868 |
| rs2253310 | 6 | 108888593 | G | C | 0.628085 | 0.017295 | 0.002485 | 3.40E-12 | 48.44748 |
| rs2292238 | 12 | 56493822 | C | A | 0.408667 | -0.0177 | 0.002448 | 4.83E-13 | 52.27931 |
| rs2307111 | 5 | 75003678 | C | T | 0.393183 | -0.02895 | 0.002462 | 6.35E-32 | 138.3022 |
| rs2318543 | 4 | 67803263 | G | A | 0.782495 | -0.0194 | 0.002922 | 3.12E-11 | 44.10116 |
| rs2384054 | 2 | 25156773 | C | T | 0.489674 | 0.035027 | 0.0024 | 3.18E-48 | 212.9834 |
| rs2398861 | 9 | 96430747 | G | A | 0.256599 | 0.022089 | 0.002761 | 1.25E-15 | 63.98957 |
| rs241460 | 1 | 49782772 | G | A | 0.68187 | -0.02054 | 0.002575 | 1.52E-15 | 63.60794 |
| rs2425857 | 20 | 44914134 | G | A | 0.554635 | -0.01359 | 0.002416 | 1.84E-08 | 31.65622 |
| rs2439823 | 10 | 99778226 | G | A | 0.547705 | 0.021636 | 0.002419 | 3.81E-19 | 79.97574 |
| rs2450254 | 11 | 69449784 | T | A | 0.415003 | -0.01407 | 0.002443 | 8.53E-09 | 33.15276 |
| rs2450445 | 10 | 93038530 | A | G | 0.323973 | -0.01537 | 0.002563 | 2.02E-09 | 35.95263 |
| rs245775 | 5 | 170532105 | G | A | 0.728826 | 0.02033 | 0.002705 | 5.65E-14 | 56.49232 |
| rs2470392 | 12 | 2155430 | C | T | 0.287428 | 0.014545 | 0.00266 | 4.54E-08 | 29.90592 |
| rs2474898 | 6 | 51769245 | T | C | 0.345803 | 0.01461 | 0.002523 | 7.03E-09 | 33.52929 |
| rs2482704 | 9 | 94182363 | T | G | 0.423422 | -0.01383 | 0.002432 | 1.30E-08 | 32.32776 |
| rs2606228 | 3 | 183537759 | C | A | 0.642769 | -0.01557 | 0.002536 | 8.29E-10 | 37.69253 |
| rs2609181 | 2 | 6161899 | C | G | 0.281859 | -0.01612 | 0.002687 | 2.00E-09 | 35.97375 |
| rs2616192 | 8 | 20668624 | T | G | 0.67249 | 0.014076 | 0.002572 | 4.45E-08 | 29.94472 |
| rs2678204 | 1 | 201800511 | G | T | 0.341813 | 0.028087 | 0.002536 | 1.65E-28 | 122.6865 |
| rs2711111 | 7 | 24529055 | G | A | 0.565043 | -0.01472 | 0.002457 | 2.12E-09 | 35.86125 |
| rs2725371 | 8 | 30854033 | G | A | 0.696308 | -0.01809 | 0.002621 | 5.12E-12 | 47.64353 |
| rs273505 | 19 | 18217147 | C | T | 0.419906 | 0.018863 | 0.002434 | 9.27E-15 | 60.05035 |
| rs2814942 | 6 | 34644261 | A | G | 0.328044 | 0.028824 | 0.002554 | 1.59E-29 | 127.337 |
| rs28366156 | 6 | 31671498 | C | T | 0.131356 | -0.029 | 0.003556 | 3.46E-16 | 66.52774 |
| rs28447555 | 3 | 107385668 | T | C | 0.180987 | 0.018508 | 0.003128 | 3.29E-09 | 35.0084 |
| rs28489620 | 22 | 41804716 | A | G | 0.290982 | -0.01473 | 0.002663 | 3.18E-08 | 30.59792 |
| rs2861685 | 2 | 67837553 | C | T | 0.411371 | -0.01765 | 0.002438 | 4.52E-13 | 52.40904 |
| rs286818 | 5 | 107433446 | A | T | 0.170248 | -0.02915 | 0.003204 | 9.41E-20 | 82.73875 |
| rs2962082 | 16 | 62816628 | A | G | 0.482549 | -0.01354 | 0.002408 | 1.88E-08 | 31.61861 |
| rs2975693 | 8 | 10114474 | C | T | 0.109281 | 0.023826 | 0.003875 | 7.85E-10 | 37.79872 |
| rs34045288 | 6 | 40369081 | T | C | 0.335903 | 0.025998 | 0.002545 | 1.72E-24 | 104.3422 |
| rs34095326 | 19 | 45395844 | A | G | 0.114134 | -0.02444 | 0.003792 | 1.16E-10 | 41.54212 |
| rs34236292 | 4 | 140786038 | T | G | 0.32772 | -0.0141 | 0.002567 | 3.96E-08 | 30.16949 |
| rs34361149 | 1 | 72750470 | A | G | 0.19259 | -0.03099 | 0.003045 | 2.50E-24 | 103.5942 |
| rs34373881 | 3 | 20432033 | A | G | 0.278276 | -0.01689 | 0.002687 | 3.28E-10 | 39.5007 |
| rs34774377 | 5 | 167001890 | C | T | 0.120337 | -0.02084 | 0.003691 | 1.63E-08 | 31.88977 |
| rs34811474 | 4 | 25408838 | A | G | 0.231875 | -0.0306 | 0.002843 | 5.30E-27 | 115.8022 |
| rs34966008 | 17 | 34919623 | T | C | 0.408148 | -0.01992 | 0.002443 | 3.59E-16 | 66.45494 |
| rs35025195 | 11 | 134522597 | A | G | 0.166132 | -0.02305 | 0.00323 | 9.68E-13 | 50.91224 |
| rs35193668 | 13 | 33092929 | T | C | 0.361915 | -0.01668 | 0.0025 | 2.55E-11 | 44.49548 |
| rs35483388 | 11 | 122545146 | T | C | 0.379617 | 0.01408 | 0.002486 | 1.48E-08 | 32.07834 |
| rs35626515 | 16 | 28649651 | A | C | 0.407965 | 0.02657 | 0.002445 | 1.71E-27 | 118.0471 |
| rs35722922 | 2 | 47002226 | G | A | 0.385007 | -0.01646 | 0.002478 | 3.08E-11 | 44.12615 |
| rs357501 | 3 | 153964496 | A | G | 0.377514 | 0.015404 | 0.002491 | 6.24E-10 | 38.24613 |
| rs35851183 | 4 | 80717182 | G | A | 0.357777 | 0.016761 | 0.002509 | 2.36E-11 | 44.6463 |
| rs35882248 | 2 | 230627955 | T | C | 0.314642 | 0.019232 | 0.002589 | 1.11E-13 | 55.1585 |
| rs36007635 | 6 | 163009335 | A | G | 0.138403 | -0.02 | 0.003481 | 9.21E-09 | 33.00309 |
| rs362307 | 4 | 3241845 | T | C | 0.076028 | 0.031354 | 0.004582 | 7.74E-12 | 46.83267 |
| rs3759584 | 14 | 103990799 | C | T | 0.361958 | -0.01658 | 0.002514 | 4.20E-11 | 43.51903 |
| rs3802858 | 11 | 115078492 | C | T | 0.427488 | -0.01647 | 0.00243 | 1.22E-11 | 45.94363 |
| rs3803286 | 14 | 103246470 | G | A | 0.666379 | -0.02079 | 0.002545 | 3.08E-16 | 66.75638 |
| rs3810291 | 19 | 47569003 | A | G | 0.677478 | 0.029685 | 0.002568 | 6.54E-31 | 133.6707 |
| rs3843540 | 7 | 99126640 | C | T | 0.148581 | -0.02417 | 0.003382 | 8.89E-13 | 51.07883 |
| rs3844598 | 5 | 140992235 | G | A | 0.528663 | 0.013699 | 0.00241 | 1.32E-08 | 32.307 |
| rs3861879 | 9 | 129464856 | G | A | 0.43683 | 0.013735 | 0.002431 | 1.62E-08 | 31.91142 |
| rs3897102 | 12 | 123492112 | T | C | 0.412248 | 0.014726 | 0.00246 | 2.14E-09 | 35.84765 |
| rs390192 | 6 | 83359135 | G | A | 0.522445 | -0.01544 | 0.002421 | 1.78E-10 | 40.69308 |
| rs4246657 | 2 | 229006134 | T | C | 0.340914 | 0.017006 | 0.002534 | 1.94E-11 | 45.02967 |
| rs4261944 | 4 | 31003636 | G | T | 0.362956 | 0.015489 | 0.002504 | 6.22E-10 | 38.25237 |
| rs4402589 | 16 | 29954654 | G | T | 0.55278 | 0.029152 | 0.002417 | 1.68E-33 | 145.5159 |
| rs4467770 | 6 | 12086826 | A | G | 0.730668 | 0.015669 | 0.002717 | 8.07E-09 | 33.26069 |
| rs4474229 | 1 | 195037001 | A | G | 0.372338 | -0.01588 | 0.002483 | 1.62E-10 | 40.87821 |
| rs4482463 | 2 | 205375909 | A | C | 0.924181 | -0.03409 | 0.004545 | 6.43E-14 | 56.23919 |
| rs4502882 | 5 | 153093998 | T | C | 0.656893 | -0.01472 | 0.002529 | 5.91E-09 | 33.86659 |
| rs4595495 | 10 | 53673286 | G | A | 0.422607 | 0.013738 | 0.002436 | 1.70E-08 | 31.81708 |
| rs4648450 | 1 | 2723214 | A | C | 0.466133 | -0.01544 | 0.002422 | 1.85E-10 | 40.62193 |
| rs4671328 | 2 | 58935282 | G | T | 0.552555 | -0.02177 | 0.002435 | 3.84E-19 | 79.959 |
| rs4687770 | 3 | 51755065 | C | T | 0.133375 | -0.01934 | 0.003535 | 4.50E-08 | 29.9205 |
| rs4718964 | 7 | 70038969 | T | G | 0.412194 | 0.014565 | 0.002447 | 2.64E-09 | 35.43606 |
| rs4757144 | 11 | 13331226 | A | G | 0.593047 | 0.016524 | 0.002446 | 1.42E-11 | 45.64264 |
| rs4776970 | 15 | 68080886 | T | A | 0.35707 | -0.02546 | 0.002505 | 2.89E-24 | 103.3114 |
| rs4777541 | 15 | 73082240 | T | C | 0.765041 | 0.019651 | 0.002846 | 5.08E-12 | 47.65947 |
| rs4778918 | 15 | 79434040 | C | T | 0.422703 | -0.0152 | 0.002432 | 4.11E-10 | 39.0643 |
| rs4790841 | 17 | 1835482 | T | C | 0.154867 | -0.02952 | 0.003329 | 7.49E-19 | 78.63931 |
| rs487662 | 11 | 64481880 | A | G | 0.753235 | 0.015437 | 0.00279 | 3.14E-08 | 30.6222 |
| rs4911382 | 20 | 32553095 | T | C | 0.584238 | 0.015559 | 0.002443 | 1.90E-10 | 40.56604 |
| rs491711 | 11 | 28742220 | C | A | 0.310988 | -0.01584 | 0.002609 | 1.28E-09 | 36.85153 |
| rs4921301 | 5 | 159984492 | T | C | 0.209817 | -0.01822 | 0.002984 | 1.02E-09 | 37.29505 |
| rs4929923 | 11 | 8639200 | C | T | 0.647328 | 0.017384 | 0.002511 | 4.39E-12 | 47.94427 |
| rs525101 | 13 | 111969728 | C | T | 0.371963 | 0.016496 | 0.00249 | 3.50E-11 | 43.87695 |
| rs539515 | 1 | 177889025 | C | A | 0.207529 | 0.047487 | 0.00296 | 6.64E-58 | 257.402 |
| rs550974 | 11 | 118937106 | T | A | 0.404297 | 0.01589 | 0.002448 | 8.55E-11 | 42.13159 |
| rs55689274 | 15 | 47758909 | A | G | 0.285711 | -0.01483 | 0.002663 | 2.53E-08 | 31.0378 |
| rs55726687 | 12 | 991306 | A | G | 0.211138 | 0.023863 | 0.002942 | 5.09E-16 | 65.76938 |
| rs55886426 | 20 | 62567684 | G | C | 0.055432 | -0.03164 | 0.005519 | 9.86E-09 | 32.87097 |
| rs55938344 | 12 | 108310327 | C | A | 0.242702 | -0.01682 | 0.002818 | 2.39E-09 | 35.62593 |
| rs56067609 | 7 | 133582465 | T | A | 0.136594 | -0.01936 | 0.003502 | 3.21E-08 | 30.57519 |
| rs56161855 | 17 | 46288649 | T | A | 0.132008 | 0.022972 | 0.003549 | 9.65E-11 | 41.89398 |
| rs56212061 | 19 | 19394640 | T | C | 0.149969 | -0.02111 | 0.003373 | 3.89E-10 | 39.16929 |
| rs56773984 | 2 | 220177285 | T | A | 0.16742 | -0.01792 | 0.003232 | 2.96E-08 | 30.73754 |
| rs56803094 | 15 | 99222509 | G | A | 0.227111 | -0.01888 | 0.002874 | 5.04E-11 | 43.16273 |
| rs57636386 | 18 | 58048295 | C | T | 0.083415 | -0.04277 | 0.004353 | 8.92E-23 | 96.51386 |
| rs58862095 | 7 | 75081418 | T | C | 0.419757 | -0.02438 | 0.00244 | 1.65E-23 | 99.86151 |
| rs588660 | 1 | 96886604 | A | G | 0.585856 | 0.018301 | 0.002441 | 6.52E-14 | 56.2136 |
| rs59104534 | 8 | 25666169 | T | C | 0.301112 | 0.014978 | 0.002629 | 1.22E-08 | 32.45848 |
| rs5995843 | 22 | 40697377 | G | A | 0.348993 | -0.01744 | 0.002519 | 4.43E-12 | 47.92899 |
| rs6050446 | 20 | 25195509 | G | A | 0.966803 | 0.042077 | 0.00676 | 4.83E-10 | 38.74574 |
| rs60654199 | 3 | 141267295 | A | C | 0.066648 | 0.031266 | 0.00481 | 8.03E-11 | 42.25361 |
| rs60764613 | 18 | 1839911 | T | G | 0.144857 | 0.023587 | 0.003426 | 5.84E-12 | 47.38672 |
| rs61813324 | 1 | 156049877 | T | C | 0.134883 | 0.027919 | 0.003563 | 4.69E-15 | 61.39381 |
| rs61826867 | 1 | 174377435 | G | A | 0.109772 | 0.025135 | 0.003842 | 6.05E-11 | 42.80582 |
| rs61871615 | 10 | 102487140 | T | C | 0.090523 | -0.02803 | 0.004373 | 1.47E-10 | 41.06813 |
| rs61903695 | 11 | 89922417 | G | A | 0.255999 | 0.016359 | 0.002752 | 2.78E-09 | 35.33352 |
| rs61969510 | 13 | 86484025 | C | T | 0.284412 | 0.015533 | 0.002695 | 8.25E-09 | 33.21766 |
| rs62106258 | 2 | 417167 | C | T | 0.048467 | -0.09069 | 0.005592 | 3.93E-59 | 263.0419 |
| rs62147189 | 2 | 86807853 | G | T | 0.622 | -0.01701 | 0.002513 | 1.32E-11 | 45.79252 |
| rs62246314 | 3 | 9504099 | A | G | 0.101276 | 0.0228 | 0.003981 | 1.02E-08 | 32.80709 |
| rs62407562 | 6 | 33530346 | A | T | 0.268967 | 0.017085 | 0.002709 | 2.87E-10 | 39.76183 |
| rs62543438 | 9 | 73896870 | C | G | 0.271966 | -0.0148 | 0.0027 | 4.22E-08 | 30.04781 |
| rs6265 | 11 | 27679916 | T | C | 0.189726 | -0.04023 | 0.003062 | 2.04E-39 | 172.6081 |
| rs6536575 | 4 | 162091639 | C | T | 0.518496 | 0.014062 | 0.002405 | 4.97E-09 | 34.20166 |
| rs6575340 | 14 | 94023972 | A | G | 0.637416 | 0.022878 | 0.002504 | 6.48E-20 | 83.4762 |
| rs6601527 | 8 | 10665444 | A | C | 0.587819 | -0.02174 | 0.002445 | 6.18E-19 | 79.01937 |
| rs66679256 | 4 | 18351898 | T | C | 0.446142 | 0.016297 | 0.002422 | 1.70E-11 | 45.29065 |
| rs6687953 | 1 | 112293512 | G | A | 0.391351 | 0.015895 | 0.002461 | 1.06E-10 | 41.70321 |
| rs66922415 | 18 | 57848651 | G | A | 0.234362 | 0.052096 | 0.002832 | 1.55E-75 | 338.4007 |
| rs6705567 | 2 | 55320173 | C | T | 0.3788 | -0.01362 | 0.002495 | 4.79E-08 | 29.80145 |
| rs6722241 | 2 | 100803778 | C | T | 0.270908 | -0.02017 | 0.002714 | 1.06E-13 | 55.25462 |
| rs6739755 | 2 | 59330227 | G | A | 0.602976 | -0.02096 | 0.00246 | 1.62E-17 | 72.56405 |
| rs67609008 | 10 | 126640936 | C | T | 0.283597 | 0.014601 | 0.002674 | 4.78E-08 | 29.80612 |
| rs6780459 | 3 | 104624105 | T | A | 0.746681 | 0.01636 | 0.002762 | 3.18E-09 | 35.07515 |
| rs67844506 | 20 | 51103807 | G | A | 0.183242 | -0.02592 | 0.003109 | 7.65E-17 | 69.50451 |
| rs6789488 | 3 | 136146030 | C | T | 0.750784 | 0.020009 | 0.002777 | 5.85E-13 | 51.90174 |
| rs6809307 | 3 | 156862041 | T | C | 0.25622 | 0.015707 | 0.00276 | 1.27E-08 | 32.3799 |
| rs6831020 | 4 | 55500226 | A | C | 0.295229 | -0.01594 | 0.002631 | 1.39E-09 | 36.69156 |
| rs6861649 | 5 | 50864788 | C | T | 0.607485 | 0.014348 | 0.002469 | 6.23E-09 | 33.76147 |
| rs6950388 | 7 | 1270699 | A | G | 0.795004 | 0.017228 | 0.002976 | 7.10E-09 | 33.50928 |
| rs7006178 | 8 | 14252384 | C | G | 0.287875 | 0.015504 | 0.002659 | 5.50E-09 | 34.00566 |
| rs7030732 | 9 | 124630006 | A | C | 0.607889 | -0.01516 | 0.002459 | 7.04E-10 | 38.01044 |
| rs704061 | 12 | 89771903 | C | T | 0.452703 | 0.01587 | 0.002414 | 4.88E-11 | 43.22553 |
| rs7094644 | 10 | 16773864 | A | G | 0.674176 | 0.015282 | 0.002611 | 4.81E-09 | 34.26774 |
| rs7116641 | 11 | 43696917 | G | T | 0.316797 | 0.024422 | 0.002586 | 3.67E-21 | 89.1553 |
| rs7124681 | 11 | 47529947 | A | C | 0.408057 | 0.026746 | 0.00244 | 5.90E-28 | 120.1587 |
| rs7132908 | 12 | 50263148 | A | G | 0.38392 | 0.028534 | 0.002472 | 8.27E-31 | 133.203 |
| rs7138383 | 12 | 103724090 | A | G | 0.253485 | -0.02138 | 0.002765 | 1.05E-14 | 59.79833 |
| rs7141420 | 14 | 79899454 | T | C | 0.514078 | 0.020815 | 0.002419 | 7.74E-18 | 74.02761 |
| rs71495049 | 10 | 34014435 | A | G | 0.084031 | 0.02655 | 0.00433 | 8.69E-10 | 37.60176 |
| rs7183417 | 15 | 46586088 | T | C | 0.432569 | 0.0149 | 0.002428 | 8.37E-10 | 37.67348 |
| rs7189149 | 16 | 69144151 | G | C | 0.127107 | 0.021347 | 0.003608 | 3.29E-09 | 35.00597 |
| rs7195386 | 16 | 24578458 | C | T | 0.506441 | -0.01588 | 0.002407 | 4.21E-11 | 43.51668 |
| rs7201895 | 16 | 407723 | A | G | 0.355984 | -0.01734 | 0.002525 | 6.54E-12 | 47.16216 |
| rs7218014 | 17 | 65832016 | C | T | 0.196595 | 0.020699 | 0.003027 | 8.05E-12 | 46.7567 |
| rs72697614 | 1 | 107514107 | A | C | 0.320396 | 0.014951 | 0.002606 | 9.67E-09 | 32.90827 |
| rs72820274 | 2 | 104412924 | A | G | 0.41876 | 0.016002 | 0.002439 | 5.39E-11 | 43.03376 |
| rs72892910 | 6 | 50816887 | T | G | 0.170442 | 0.040166 | 0.003204 | 4.89E-36 | 157.1294 |
| rs72976986 | 19 | 4050424 | A | G | 0.191187 | -0.02405 | 0.003088 | 6.81E-15 | 60.65822 |
| rs73050254 | 7 | 3129614 | A | G | 0.136682 | 0.019322 | 0.003502 | 3.45E-08 | 30.43995 |
| rs73144053 | 3 | 88085826 | A | C | 0.329919 | -0.01536 | 0.00256 | 2.01E-09 | 35.96692 |
| rs73169730 | 3 | 170734438 | G | A | 0.276291 | 0.019463 | 0.002687 | 4.35E-13 | 52.4819 |
| rs7321331 | 13 | 31015138 | A | G | 0.741132 | 0.017555 | 0.002755 | 1.87E-10 | 40.59735 |
| rs73213484 | 4 | 28489339 | T | A | 0.139056 | -0.0212 | 0.003474 | 1.05E-09 | 37.22678 |
| rs7331420 | 13 | 99236471 | A | G | 0.284027 | -0.01473 | 0.00267 | 3.44E-08 | 30.44309 |
| rs7442885 | 5 | 87682877 | G | C | 0.210104 | -0.02543 | 0.002957 | 7.96E-18 | 73.97136 |
| rs7498044 | 15 | 92573639 | A | G | 0.218661 | -0.01765 | 0.002932 | 1.75E-09 | 36.23293 |
| rs750090 | 4 | 152931436 | C | T | 0.356153 | -0.01563 | 0.002535 | 6.93E-10 | 38.04123 |
| rs752179 | 3 | 108024070 | A | T | 0.301702 | -0.01437 | 0.002621 | 4.17E-08 | 30.07006 |
| rs75499503 | 6 | 26145217 | T | C | 0.219955 | -0.01978 | 0.002946 | 1.88E-11 | 45.09473 |
| rs7553158 | 1 | 75005238 | A | G | 0.562389 | -0.01742 | 0.002424 | 6.70E-13 | 51.63391 |
| rs75557510 | 3 | 84321469 | G | A | 0.061496 | -0.03779 | 0.005104 | 1.33E-13 | 54.81237 |
| rs756717 | 16 | 72996162 | A | G | 0.398859 | -0.01467 | 0.002482 | 3.38E-09 | 34.95425 |
| rs76040172 | 21 | 46488959 | A | G | 0.05444 | -0.04076 | 0.005308 | 1.62E-14 | 58.95047 |
| rs7701777 | 5 | 27178172 | G | T | 0.282759 | -0.01703 | 0.002668 | 1.74E-10 | 40.74041 |
| rs7719067 | 5 | 153538241 | G | A | 0.573835 | -0.0162 | 0.002426 | 2.41E-11 | 44.60636 |
| rs7723426 | 5 | 43186097 | C | T | 0.675017 | 0.014669 | 0.002566 | 1.09E-08 | 32.68151 |
| rs7755574 | 6 | 153364643 | T | G | 0.281153 | 0.015496 | 0.002674 | 6.87E-09 | 33.57304 |
| rs7774 | 17 | 4801163 | A | C | 0.309138 | 0.016634 | 0.002615 | 2.01E-10 | 40.45518 |
| rs778094 | 2 | 147903802 | A | G | 0.577568 | -0.01485 | 0.002437 | 1.09E-09 | 37.15749 |
| rs7852189 | 9 | 103121547 | G | A | 0.311364 | 0.016554 | 0.002589 | 1.61E-10 | 40.88842 |
| rs78565420 | 8 | 85703065 | T | C | 0.054186 | 0.033731 | 0.005454 | 6.24E-10 | 38.24627 |
| rs79113395 | 1 | 1590521 | A | G | 0.265449 | -0.02091 | 0.002732 | 1.95E-14 | 58.59096 |
| rs7933085 | 11 | 130796248 | G | A | 0.509195 | 0.015739 | 0.002415 | 7.21E-11 | 42.46469 |
| rs7941828 | 11 | 30430331 | T | C | 0.360093 | -0.0156 | 0.0025 | 4.34E-10 | 38.95768 |
| rs7952102 | 11 | 892089 | C | T | 0.387339 | -0.01523 | 0.002465 | 6.59E-10 | 38.14076 |
| rs7992832 | 13 | 28013501 | T | C | 0.276544 | -0.01756 | 0.002692 | 7.02E-11 | 42.51724 |
| rs799449 | 7 | 44784697 | T | C | 0.558481 | 0.019902 | 0.002427 | 2.41E-16 | 67.24461 |
| rs8015400 | 14 | 25930988 | A | C | 0.678151 | 0.021088 | 0.002572 | 2.45E-16 | 67.21029 |
| rs80330591 | 2 | 159368720 | A | G | 0.146844 | -0.02136 | 0.003393 | 3.08E-10 | 39.62414 |
| rs8078135 | 17 | 21268583 | T | C | 0.488467 | -0.0195 | 0.002412 | 6.23E-16 | 65.36947 |
| rs8087074 | 18 | 45923482 | T | G | 0.261119 | 0.017007 | 0.002748 | 6.02E-10 | 38.31584 |
| rs8134638 | 21 | 40644170 | C | T | 0.375763 | 0.015169 | 0.002481 | 9.68E-10 | 37.39042 |
| rs815163 | 1 | 190294726 | C | T | 0.561603 | -0.01825 | 0.00242 | 4.69E-14 | 56.85962 |
| rs845084 | 10 | 125220036 | A | G | 0.257551 | 0.02034 | 0.002757 | 1.61E-13 | 54.4327 |
| rs846546 | 1 | 210261609 | T | A | 0.418217 | -0.01469 | 0.002436 | 1.63E-09 | 36.3765 |
| rs862320 | 16 | 69651866 | T | C | 0.410152 | -0.02282 | 0.002446 | 1.10E-20 | 86.98576 |
| rs869400 | 3 | 185826740 | G | T | 0.815827 | 0.030726 | 0.003107 | 4.70E-23 | 97.78402 |
| rs879620 | 16 | 4015729 | T | C | 0.615276 | 0.025612 | 0.002478 | 4.93E-25 | 106.8148 |
| rs9267671 | 6 | 31880480 | A | G | 0.060402 | 0.032371 | 0.005042 | 1.36E-10 | 41.22704 |
| rs9291822 | 5 | 64076515 | T | C | 0.515455 | -0.01361 | 0.002426 | 2.00E-08 | 31.49937 |
| rs9320823 | 6 | 98429337 | C | T | 0.601901 | 0.01894 | 0.002458 | 1.30E-14 | 59.39254 |
| rs9342196 | 6 | 90124635 | T | C | 0.189501 | 0.016733 | 0.003066 | 4.83E-08 | 29.78377 |
| rs935166 | 2 | 26949366 | A | G | 0.506935 | -0.01549 | 0.002403 | 1.13E-10 | 41.58162 |
| rs9402104 | 6 | 129410736 | A | G | 0.584134 | 0.013902 | 0.002452 | 1.43E-08 | 32.14605 |
| rs946185 | 10 | 76423739 | G | A | 0.593276 | -0.01469 | 0.002462 | 2.41E-09 | 35.61381 |
| rs9515455 | 13 | 112239671 | A | G | 0.416606 | 0.017964 | 0.00245 | 2.25E-13 | 53.7767 |
| rs9527906 | 13 | 59403551 | A | G | 0.762395 | -0.01618 | 0.002834 | 1.13E-08 | 32.60992 |
| rs9641499 | 7 | 112984493 | A | C | 0.434095 | -0.0171 | 0.002423 | 1.68E-12 | 49.83371 |
| rs9688977 | 6 | 154336892 | C | T | 0.146486 | 0.023978 | 0.003406 | 1.93E-12 | 49.55796 |
| rs9843653 | 3 | 49920571 | C | T | 0.514555 | 0.031726 | 0.002404 | 9.35E-40 | 174.1592 |
| rs9847186 | 3 | 25081857 | A | G | 0.427737 | -0.01428 | 0.002434 | 4.36E-09 | 34.45813 |

Supplementary Table S4. Identification of 78 BMI-related SNPs selected as IVs for BD

| **SNP** | **Chr** | **Position** | **EA** | **OA** | **EAF** | **β** | **SE** | ***p*** | **F** |
| --- | --- | --- | --- | --- | --- | --- | --- | --- | --- |
| rs10179482 | 2 | 81004626 | A | G | 0.485465 | 0.005773 | 0.001007 | 9.90E-09 | 32.8559 |
| rs10212155 | 3 | 117785505 | A | G | 0.852761 | 0.010355 | 0.001423 | 3.40E-13 | 52.97429 |
| rs10233018 | 7 | 117523709 | G | A | 0.503991 | 0.007789 | 0.001005 | 9.20E-15 | 60.06879 |
| rs10774625 | 12 | 111910219 | G | A | 0.503725 | -0.00659 | 0.001003 | 5.00E-11 | 43.15946 |
| rs10863714 | 1 | 208710593 | G | A | 0.576592 | -0.00582 | 0.001016 | 1.00E-08 | 32.77603 |
| rs10952199 | 7 | 1665339 | T | C | 0.426753 | -0.00687 | 0.001017 | 1.40E-11 | 45.68043 |
| rs10956808 | 8 | 92775372 | G | T | 0.422058 | -0.00671 | 0.00102 | 4.70E-11 | 43.31263 |
| rs10988799 | 9 | 102158106 | T | C | 0.528587 | 0.005947 | 0.001008 | 3.60E-09 | 34.82406 |
| rs11165623 | 1 | 96893000 | A | G | 0.504402 | 0.005496 | 0.001004 | 4.40E-08 | 29.96442 |
| rs1124639 | 2 | 200775744 | C | T | 0.554782 | 0.006597 | 0.001011 | 6.70E-11 | 42.61189 |
| rs1150023 | 10 | 9360823 | C | A | 0.737332 | -0.00645 | 0.001143 | 1.60E-08 | 31.88336 |
| rs1174864 | 7 | 53127559 | A | G | 0.550251 | 0.006206 | 0.001011 | 8.40E-10 | 37.66488 |
| rs12209519 | 6 | 67549140 | G | A | 0.408089 | 0.005691 | 0.001026 | 2.90E-08 | 30.75892 |
| rs12244388 | 10 | 104640052 | A | G | 0.338891 | 0.008567 | 0.001061 | 6.80E-16 | 65.18937 |
| rs12272735 | 11 | 7950024 | G | A | 0.392103 | 0.005781 | 0.001027 | 1.80E-08 | 31.66948 |
| rs12333760 | 7 | 99185406 | C | T | 0.165187 | -0.00828 | 0.001355 | 9.90E-10 | 37.34006 |
| rs12450028 | 17 | 2207425 | T | C | 0.34525 | 0.006131 | 0.001055 | 6.30E-09 | 33.73906 |
| rs1246265 | 9 | 86761745 | C | T | 0.69544 | 0.006069 | 0.001094 | 2.90E-08 | 30.79657 |
| rs12902636 | 15 | 47971451 | T | C | 0.502529 | -0.00594 | 0.001008 | 3.70E-09 | 34.77866 |
| rs13162305 | 5 | 12122698 | T | A | 0.335129 | 0.00599 | 0.001071 | 2.20E-08 | 31.28364 |
| rs1322525 | 6 | 19093152 | G | A | 0.36713 | 0.006389 | 0.001043 | 9.10E-10 | 37.50588 |
| rs1324481 | 1 | 33892964 | G | T | 0.681417 | 0.006897 | 0.001079 | 1.60E-10 | 40.87231 |
| rs1363101 | 5 | 103941070 | G | T | 0.520698 | -0.00577 | 0.001005 | 9.30E-09 | 32.97522 |
| rs1373178 | 18 | 49967811 | G | T | 0.594204 | -0.00625 | 0.001027 | 1.20E-09 | 36.96165 |
| rs150294 | 15 | 89931148 | G | A | 0.401317 | -0.00755 | 0.001029 | 2.10E-13 | 53.90795 |
| rs1549212 | 5 | 166996722 | T | C | 0.626251 | -0.00663 | 0.001039 | 1.80E-10 | 40.7145 |
| rs1718705 | 3 | 61823653 | C | G | 0.317331 | 0.006462 | 0.001084 | 2.50E-09 | 35.52037 |
| rs17584022 | 11 | 59190650 | A | G | 0.288018 | -0.00659 | 0.001109 | 2.70E-09 | 35.36412 |
| rs1876066 | 10 | 10040774 | C | T | 0.497781 | -0.00569 | 0.001014 | 1.90E-08 | 31.56592 |
| rs1899896 | 8 | 93201036 | T | C | 0.297037 | 0.007233 | 0.001103 | 5.50E-11 | 42.99655 |
| rs2155292 | 11 | 112910783 | G | A | 0.387722 | 0.014746 | 0.001031 | 2.00E-46 | 204.6931 |
| rs2175207 | 10 | 87357738 | G | A | 0.159486 | 0.007604 | 0.001371 | 2.90E-08 | 30.75037 |
| rs2183573 | 21 | 40574305 | G | A | 0.572269 | -0.00584 | 0.001016 | 9.20E-09 | 32.99727 |
| rs28809490 | 22 | 46414043 | A | G | 0.309077 | -0.00633 | 0.001088 | 5.90E-09 | 33.86212 |
| rs303753 | 18 | 21074922 | A | G | 0.346408 | -0.00608 | 0.001064 | 1.10E-08 | 32.60045 |
| rs34335016 | 1 | 154144681 | T | A | 0.12052 | -0.00864 | 0.001543 | 2.20E-08 | 31.32044 |
| rs35498642 | 3 | 85928634 | T | C | 0.392512 | -0.00624 | 0.001028 | 1.30E-09 | 36.87424 |
| rs35892365 | 8 | 26280685 | T | C | 0.243677 | -0.00671 | 0.001196 | 2.00E-08 | 31.47734 |
| rs3783177 | 13 | 101151360 | G | T | 0.238285 | -0.00729 | 0.001183 | 7.30E-10 | 37.93089 |
| rs3790286 | 20 | 19655938 | C | T | 0.542753 | 0.005591 | 0.001013 | 3.40E-08 | 30.47207 |
| rs41513151 | 7 | 121962809 | A | G | 0.216228 | 0.007256 | 0.001221 | 2.80E-09 | 35.31086 |
| rs4422110 | 2 | 146114898 | T | C | 0.534619 | -0.00884 | 0.001007 | 1.50E-18 | 77.21296 |
| rs465646 | 6 | 111620758 | A | G | 0.84077 | -0.01174 | 0.001374 | 1.30E-17 | 72.96613 |
| rs4680392 | 3 | 157404329 | C | T | 0.673873 | 0.006157 | 0.001073 | 9.60E-09 | 32.91323 |
| rs4856598 | 3 | 85650790 | C | A | 0.62769 | -0.00839 | 0.001049 | 1.20E-15 | 64.06486 |
| rs529206 | 11 | 132213460 | T | C | 0.589177 | -0.00585 | 0.001032 | 1.40E-08 | 32.18 |
| rs55864295 | 4 | 70512801 | G | A | 0.185129 | -0.00737 | 0.001291 | 1.10E-08 | 32.62304 |
| rs56166763 | 4 | 140943169 | C | G | 0.368511 | -0.00582 | 0.001041 | 2.30E-08 | 31.2637 |
| rs58400863 | 4 | 31184484 | A | G | 0.341099 | -0.00633 | 0.001064 | 2.70E-09 | 35.35736 |
| rs61785503 | 1 | 50458443 | T | C | 0.23512 | 0.007048 | 0.001183 | 2.50E-09 | 35.52173 |
| rs6265 | 11 | 27679916 | T | C | 0.18847 | -0.00838 | 0.001284 | 6.70E-11 | 42.6048 |
| rs6438208 | 3 | 114170272 | A | G | 0.25141 | -0.00656 | 0.001201 | 4.80E-08 | 29.81511 |
| rs6499595 | 16 | 72934443 | C | G | 0.397268 | -0.00654 | 0.001026 | 1.80E-10 | 40.64059 |
| rs67716713 | 2 | 104267572 | A | C | 0.488327 | -0.00729 | 0.001004 | 4.00E-13 | 52.66509 |
| rs7014143 | 8 | 21587997 | C | A | 0.586417 | 0.006029 | 0.00108 | 2.40E-08 | 31.1518 |
| rs7024687 | 9 | 3174579 | G | A | 0.522154 | -0.00583 | 0.001017 | 1.00E-08 | 32.83601 |
| rs71580759 | 5 | 87770123 | C | T | 0.222323 | -0.00681 | 0.001208 | 1.70E-08 | 31.77684 |
| rs7162423 | 15 | 74052756 | T | C | 0.444196 | -0.00562 | 0.00101 | 2.70E-08 | 30.8948 |
| rs7216173 | 17 | 51891405 | T | A | 0.781979 | -0.00719 | 0.001233 | 5.60E-09 | 33.98522 |
| rs72706955 | 9 | 23820210 | T | C | 0.150042 | -0.00771 | 0.001406 | 4.20E-08 | 30.06384 |
| rs73058737 | 3 | 34532026 | T | G | 0.288033 | 0.006156 | 0.001114 | 3.30E-08 | 30.54651 |
| rs75174460 | 12 | 133486532 | C | T | 0.075101 | 0.010777 | 0.001926 | 2.20E-08 | 31.3032 |
| rs7572027 | 2 | 226286009 | T | C | 0.81255 | 0.007096 | 0.001285 | 3.40E-08 | 30.47902 |
| rs75919030 | 17 | 50193197 | C | T | 0.262188 | -0.0068 | 0.001146 | 2.90E-09 | 35.22657 |
| rs763053 | 16 | 735921 | C | T | 0.225654 | -0.00842 | 0.001206 | 2.90E-12 | 48.72674 |
| rs7758291 | 6 | 129348438 | C | A | 0.684258 | 0.006023 | 0.00108 | 2.40E-08 | 31.1135 |
| rs77878475 | 16 | 18058548 | A | T | 0.084235 | -0.01167 | 0.001875 | 4.90E-10 | 38.72494 |
| rs7870475 | 9 | 128134034 | C | T | 0.475284 | 0.005659 | 0.001005 | 1.80E-08 | 31.68697 |
| rs7901348 | 10 | 63679281 | G | T | 0.551902 | -0.00603 | 0.001017 | 3.10E-09 | 35.12575 |
| rs7969559 | 12 | 69655167 | G | A | 0.720249 | -0.00622 | 0.001118 | 2.60E-08 | 30.97396 |
| rs899632 | 4 | 57749347 | C | T | 0.389049 | -0.00703 | 0.001033 | 9.90E-12 | 46.33967 |
| rs904592 | 3 | 25193102 | T | C | 0.482639 | 0.005692 | 0.001013 | 1.90E-08 | 31.56026 |
| rs905871 | 11 | 4661285 | G | A | 0.339517 | -0.00584 | 0.001061 | 3.70E-08 | 30.28003 |
| rs9375371 | 6 | 98751680 | A | G | 0.269366 | 0.007068 | 0.001134 | 4.60E-10 | 38.82325 |
| rs9423279 | 10 | 125680419 | G | C | 0.656563 | -0.00627 | 0.001078 | 5.90E-09 | 33.88344 |
| rs9597810 | 13 | 59264524 | G | C | 0.327261 | -0.00587 | 0.001072 | 4.30E-08 | 30.01478 |
| rs9835772 | 3 | 85766025 | T | A | 0.243671 | 0.006562 | 0.001169 | 2.00E-08 | 31.48492 |
| rs9845144 | 3 | 184056716 | A | G | 0.272671 | 0.006558 | 0.00114 | 8.80E-09 | 33.088 |

Supplementary Table S5. Identification of 38 BMI-related SNPs selected as IVs for BD

| **SNP** | **Chr** | **Position** | **EA** | **OA** | **EAF** | **β** | **SE** | ***p*** | **F** |
| --- | --- | --- | --- | --- | --- | --- | --- | --- | --- |
| rs10838708 | 11 | 47441513 | A | G | 0.45899 | -0.00948 | 0.001503 | 2.90E-10 | 39.76959 |
| rs11097861 | 4 | 105330133 | G | A | 0.716256 | 0.010044 | 0.001649 | 1.10E-09 | 37.07694 |
| rs11152363 | 18 | 53057188 | A | G | 0.186319 | 0.015639 | 0.001925 | 4.50E-16 | 65.9921 |
| rs113851554 | 2 | 66750564 | T | G | 0.057291 | 0.04678 | 0.003313 | 2.90E-45 | 199.345 |
| rs11635495 | 15 | 67804682 | C | T | 0.512179 | 0.009373 | 0.001485 | 2.80E-10 | 39.83737 |
| rs11790060 | 9 | 96202932 | C | T | 0.330834 | -0.01034 | 0.001579 | 5.80E-11 | 42.8997 |
| rs12049261 | 1 | 107190407 | C | G | 0.292534 | 0.011187 | 0.00163 | 6.80E-12 | 47.07397 |
| rs12470989 | 2 | 200955823 | G | A | 0.203934 | -0.01024 | 0.001845 | 2.80E-08 | 30.82647 |
| rs1430205 | 5 | 87678585 | T | C | 0.461507 | 0.009475 | 0.001491 | 2.10E-10 | 40.37904 |
| rs1547630 | 13 | 112642528 | A | G | 0.651513 | 0.009108 | 0.001564 | 5.80E-09 | 33.89416 |
| rs1592757 | 5 | 103889998 | C | G | 0.355788 | 0.010222 | 0.00155 | 4.30E-11 | 43.49074 |
| rs17151854 | 8 | 10236559 | T | G | 0.15241 | 0.012989 | 0.002074 | 3.80E-10 | 39.21133 |
| rs17709610 | 10 | 104250278 | G | A | 0.297979 | -0.00992 | 0.001621 | 9.50E-10 | 37.43119 |
| rs1988337 | 4 | 91292200 | G | A | 0.552395 | 0.008387 | 0.001496 | 2.10E-08 | 31.41346 |
| rs2014830 | 3 | 50172397 | T | C | 0.303519 | -0.0116 | 0.001623 | 8.90E-13 | 51.08027 |
| rs2062113 | 16 | 59476179 | C | T | 0.568257 | -0.00962 | 0.001503 | 1.60E-10 | 40.95302 |
| rs224032 | 10 | 64521829 | A | G | 0.550358 | 0.008391 | 0.001491 | 1.80E-08 | 31.66579 |
| rs2297787 | 10 | 104685299 | A | T | 0.080193 | -0.0178 | 0.00275 | 9.60E-11 | 41.89789 |
| rs2604551 | 4 | 15093831 | G | T | 0.640384 | -0.00848 | 0.001552 | 4.70E-08 | 29.84569 |
| rs314280 | 6 | 105400837 | G | A | 0.547047 | 0.009714 | 0.001491 | 7.30E-11 | 42.42382 |
| rs324017 | 12 | 57490100 | C | A | 0.705433 | -0.00988 | 0.001631 | 1.40E-09 | 36.6931 |
| rs4572538 | 2 | 147423012 | T | C | 0.364055 | -0.00961 | 0.001562 | 7.70E-10 | 37.83872 |
| rs4886860 | 15 | 74340336 | C | G | 0.767408 | -0.0118 | 0.001756 | 1.80E-11 | 45.14282 |
| rs56093896 | 2 | 114103966 | A | C | 0.214053 | -0.01241 | 0.001814 | 7.70E-12 | 46.83557 |
| rs56330606 | 19 | 37673953 | G | A | 0.378954 | 0.009309 | 0.00153 | 1.20E-09 | 37.00013 |
| rs56365214 | 2 | 58930167 | A | C | 0.155781 | -0.01479 | 0.002052 | 5.60E-13 | 51.97059 |
| rs6561715 | 13 | 53888526 | A | T | 0.630662 | -0.01162 | 0.001542 | 4.80E-14 | 56.79057 |
| rs6690017 | 1 | 57842729 | G | T | 0.408855 | -0.01027 | 0.00151 | 1.10E-11 | 46.21663 |
| rs68094047 | 12 | 109855201 | T | C | 0.251267 | 0.010336 | 0.001717 | 1.70E-09 | 36.24788 |
| rs6975972 | 7 | 1070468 | G | A | 0.578726 | -0.00902 | 0.001504 | 2.00E-09 | 35.95997 |
| rs705219 | 3 | 117702270 | A | T | 0.887373 | 0.013423 | 0.002353 | 1.20E-08 | 32.54311 |
| rs72924721 | 11 | 65585990 | T | C | 0.073137 | 0.016478 | 0.002881 | 1.10E-08 | 32.71248 |
| rs7711696 | 5 | 135486536 | T | G | 0.305042 | 0.011172 | 0.001611 | 4.10E-12 | 48.06565 |
| rs931221 | 12 | 84727318 | A | T | 0.236738 | 0.010636 | 0.001753 | 1.30E-09 | 36.80752 |
| rs9570080 | 13 | 59832775 | C | T | 0.344131 | -0.01064 | 0.001579 | 1.60E-11 | 45.41284 |
| rs9845387 | 3 | 116425935 | A | C | 0.040279 | -0.02186 | 0.003776 | 7.10E-09 | 33.49461 |
| rs9894577 | 17 | 43223292 | A | G | 0.3182 | 0.013205 | 0.001597 | 1.30E-16 | 68.38239 |
| rs9906181 | 17 | 21297686 | G | A | 0.687567 | -0.00915 | 0.001639 | 2.40E-08 | 31.1798 |

Supplementary Table S6. Identification of 297 BMI-related SNPs selected as IVs for BD

| **SNP** | **Chr** | **Position** | **EA** | **OA** | **EAF** | **β** | **SE** | ***p*** | **F** |
| --- | --- | --- | --- | --- | --- | --- | --- | --- | --- |
| rs10100245 | 8 | 77226919 | A | G | 0.56644 | 0.020637 | 0.002423 | 1.64E-17 | 72.53998 |
| rs10144067 | 14 | 93885198 | T | C | 0.59159 | 0.019838 | 0.002463 | 7.92E-16 | 64.89545 |
| rs10185199 | 2 | 40282202 | A | G | 0.27894 | -0.01871 | 0.002749 | 1.01E-11 | 46.3119 |
| rs10187101 | 2 | 50742227 | T | C | 0.359822 | -0.01571 | 0.002505 | 3.54E-10 | 39.3555 |
| rs10404726 | 19 | 18834514 | T | C | 0.466784 | -0.01986 | 0.002407 | 1.60E-16 | 68.05188 |
| rs10465231 | 9 | 92183413 | T | C | 0.5494 | 0.01714 | 0.002427 | 1.65E-12 | 49.86364 |
| rs1064213 | 2 | 198950240 | A | G | 0.476968 | 0.016189 | 0.002406 | 1.70E-11 | 45.28867 |
| rs10749233 | 10 | 118777998 | C | G | 0.75947 | -0.01896 | 0.002848 | 2.75E-11 | 44.34695 |
| rs10788493 | 10 | 88110792 | T | C | 0.45676 | 0.013692 | 0.00242 | 1.53E-08 | 32.01386 |
| rs10803762 | 2 | 161105876 | A | G | 0.678865 | 0.015464 | 0.002582 | 2.11E-09 | 35.87415 |
| rs10805383 | 5 | 63034606 | A | G | 0.479558 | 0.016949 | 0.002409 | 1.99E-12 | 49.50236 |
| rs10865612 | 3 | 85873898 | C | T | 0.354736 | -0.02308 | 0.002511 | 3.80E-20 | 84.53157 |
| rs10898330 | 11 | 84630335 | T | C | 0.525392 | -0.01429 | 0.002417 | 3.39E-09 | 34.94931 |
| rs10938397 | 4 | 45182527 | G | A | 0.434075 | 0.028953 | 0.002429 | 9.55E-33 | 142.0675 |
| rs10995427 | 10 | 64842838 | A | G | 0.360827 | -0.01653 | 0.002516 | 5.00E-11 | 43.18012 |
| rs11012732 | 10 | 21830104 | G | A | 0.331106 | 0.02385 | 0.002555 | 1.03E-20 | 87.12295 |
| rs11078883 | 17 | 2138828 | G | C | 0.34744 | 0.015679 | 0.002532 | 5.96E-10 | 38.33534 |
| rs11084554 | 19 | 31019807 | A | G | 0.156586 | -0.02158 | 0.003304 | 6.58E-11 | 42.64196 |
| rs11099020 | 4 | 130724902 | T | C | 0.642739 | -0.01532 | 0.002512 | 1.06E-09 | 37.2054 |
| rs11150745 | 17 | 78757626 | G | A | 0.319489 | -0.02098 | 0.002579 | 4.13E-16 | 66.17944 |
| rs111640872 | 19 | 30290357 | C | G | 0.331076 | 0.020623 | 0.002559 | 7.72E-16 | 64.94699 |
| rs11223641 | 11 | 133820022 | C | T | 0.144615 | -0.0191 | 0.003432 | 2.61E-08 | 30.98062 |
| rs112520079 | 8 | 95605375 | G | T | 0.198951 | 0.020458 | 0.003014 | 1.14E-11 | 46.06922 |
| rs11264489 | 1 | 156480831 | G | A | 0.361994 | 0.014085 | 0.002507 | 1.93E-08 | 31.56273 |
| rs112693590 | 19 | 46274553 | A | G | 0.049707 | -0.03292 | 0.00568 | 6.80E-09 | 33.59166 |
| rs1127100 | 1 | 32193647 | C | T | 0.648239 | 0.016492 | 0.002522 | 6.20E-11 | 42.76012 |
| rs113182412 | 15 | 66503584 | A | G | 0.165919 | -0.01937 | 0.003284 | 3.68E-09 | 34.78633 |
| rs113230003 | 19 | 18460956 | A | G | 0.260185 | -0.01978 | 0.002759 | 7.56E-13 | 51.39759 |
| rs113603865 | 1 | 39564930 | T | C | 0.212428 | 0.01975 | 0.002957 | 2.42E-11 | 44.60028 |
| rs11515071 | 9 | 15855545 | T | C | 0.365135 | -0.02281 | 0.002506 | 8.84E-20 | 82.86212 |
| rs11642015 | 16 | 53802494 | T | C | 0.40236 | 0.072369 | 0.002447 | 6.40E-192 | 874.3937 |
| rs11650012 | 17 | 52927879 | A | T | 0.169618 | 0.018609 | 0.003218 | 7.37E-09 | 33.43519 |
| rs11655587 | 17 | 47140794 | T | C | 0.359868 | -0.01964 | 0.002511 | 5.26E-15 | 61.16698 |
| rs11742930 | 5 | 105774098 | T | C | 0.566428 | 0.014308 | 0.002429 | 3.84E-09 | 34.70479 |
| rs11757278 | 6 | 13180454 | C | T | 0.305848 | -0.01579 | 0.002609 | 1.42E-09 | 36.64304 |
| rs11761411 | 7 | 39303649 | T | C | 0.154361 | -0.01848 | 0.00334 | 3.17E-08 | 30.60168 |
| rs117632017 | 15 | 52260107 | A | G | 0.039142 | 0.036382 | 0.006447 | 1.67E-08 | 31.84932 |
| rs11782074 | 8 | 142617096 | T | G | 0.385641 | 0.014495 | 0.002507 | 7.37E-09 | 33.43542 |
| rs11856579 | 15 | 78012688 | A | G | 0.268192 | -0.01979 | 0.002711 | 2.91E-13 | 53.27422 |
| rs12024554 | 1 | 19925759 | T | C | 0.236905 | -0.01627 | 0.002827 | 8.75E-09 | 33.10169 |
| rs12042959 | 1 | 243533273 | G | A | 0.145777 | -0.02056 | 0.003425 | 1.96E-09 | 36.01878 |
| rs12049202 | 1 | 77967523 | T | C | 0.198651 | 0.021836 | 0.003008 | 3.91E-13 | 52.69047 |
| rs12140153 | 1 | 62579891 | T | G | 0.096761 | -0.03182 | 0.004168 | 2.25E-14 | 58.30603 |
| rs12144626 | 1 | 47670525 | C | T | 0.581821 | -0.01721 | 0.002444 | 1.87E-12 | 49.61626 |
| rs12477385 | 2 | 166156146 | T | G | 0.2273 | -0.01725 | 0.002876 | 1.99E-09 | 35.98089 |
| rs12479357 | 2 | 181570507 | G | A | 0.631081 | 0.017865 | 0.002499 | 8.71E-13 | 51.11822 |
| rs12614861 | 2 | 175047184 | T | G | 0.34701 | 0.015234 | 0.002522 | 1.53E-09 | 36.49208 |
| rs12622280 | 2 | 79515954 | G | T | 0.157075 | -0.0187 | 0.003305 | 1.53E-08 | 32.01071 |
| rs12662900 | 6 | 97757782 | A | T | 0.282225 | -0.01686 | 0.002673 | 2.85E-10 | 39.77773 |
| rs12679106 | 8 | 73446870 | T | G | 0.711341 | -0.02203 | 0.002665 | 1.38E-16 | 68.33847 |
| rs1286138 | 14 | 91485445 | G | T | 0.673439 | 0.018051 | 0.002564 | 1.92E-12 | 49.56318 |
| rs12877270 | 13 | 97047020 | A | G | 0.43845 | 0.016908 | 0.002439 | 4.15E-12 | 48.05751 |
| rs12881629 | 14 | 101146413 | G | A | 0.082146 | 0.023968 | 0.004366 | 4.04E-08 | 30.13188 |
| rs12885458 | 14 | 47305486 | G | T | 0.509982 | -0.01561 | 0.002404 | 8.40E-11 | 42.16455 |
| rs1296328 | 4 | 137083193 | C | A | 0.559914 | -0.01898 | 0.002431 | 5.86E-15 | 60.95502 |
| rs12977259 | 19 | 1951123 | G | A | 0.821338 | 0.018626 | 0.003161 | 3.80E-09 | 34.7264 |
| rs12992672 | 2 | 632723 | A | G | 0.828635 | 0.050781 | 0.003182 | 2.70E-57 | 254.6103 |
| rs13047416 | 21 | 40309436 | G | C | 0.375427 | -0.01364 | 0.002489 | 4.24E-08 | 30.03788 |
| rs13062093 | 3 | 35667057 | G | T | 0.366444 | 0.01706 | 0.002491 | 7.42E-12 | 46.91575 |
| rs13076052 | 3 | 44456573 | G | C | 0.277037 | 0.016077 | 0.002701 | 2.65E-09 | 35.4261 |
| rs13135092 | 4 | 103198082 | G | A | 0.083234 | 0.050055 | 0.004374 | 2.60E-30 | 130.9321 |
| rs13174863 | 5 | 139080745 | G | A | 0.148208 | 0.024979 | 0.003401 | 2.08E-13 | 53.93468 |
| rs1320903 | 3 | 131758077 | A | G | 0.318441 | 0.022008 | 0.002581 | 1.52E-17 | 72.69441 |
| rs1327259 | 6 | 51177811 | G | A | 0.38613 | -0.014 | 0.002475 | 1.54E-08 | 32.00164 |
| rs1342391 | 1 | 96285385 | T | G | 0.669963 | 0.015869 | 0.002561 | 5.83E-10 | 38.37963 |
| rs13427822 | 2 | 213414265 | G | A | 0.272052 | -0.0199 | 0.002729 | 3.05E-13 | 53.18003 |
| rs1411432 | 9 | 16728532 | C | A | 0.183895 | 0.024946 | 0.003109 | 1.02E-15 | 64.39497 |
| rs1441264 | 13 | 79580919 | A | G | 0.591866 | 0.020638 | 0.002497 | 1.39E-16 | 68.33101 |
| rs1446585 | 2 | 136407479 | G | A | 0.228006 | -0.01711 | 0.002877 | 2.71E-09 | 35.38576 |
| rs1458156 | 12 | 41887940 | T | C | 0.488264 | 0.013785 | 0.002405 | 9.99E-09 | 32.84591 |
| rs1477290 | 5 | 87988934 | C | T | 0.135362 | 0.033539 | 0.003537 | 2.51E-21 | 89.9115 |
| rs147730268 | 12 | 123024476 | T | G | 0.09053 | -0.03611 | 0.004329 | 7.38E-17 | 69.57578 |
| rs1582931 | 5 | 122657199 | A | G | 0.472057 | -0.01486 | 0.002426 | 8.97E-10 | 37.53852 |
| rs16846140 | 2 | 212292521 | G | A | 0.33821 | 0.01619 | 0.002544 | 1.98E-10 | 40.49305 |
| rs16916303 | 9 | 30823761 | G | A | 0.119234 | -0.02072 | 0.003739 | 3.01E-08 | 30.70542 |
| rs16932761 | 8 | 67202787 | A | G | 0.251546 | -0.01797 | 0.002782 | 1.04E-10 | 41.75247 |
| rs16975459 | 18 | 39548277 | C | A | 0.121305 | 0.023972 | 0.003686 | 7.84E-11 | 42.30012 |
| rs17014332 | 1 | 209520450 | C | T | 0.210609 | 0.018029 | 0.002944 | 9.16E-10 | 37.49863 |
| rs17024393 | 1 | 110154688 | C | T | 0.025799 | 0.067508 | 0.007598 | 6.45E-19 | 78.93554 |
| rs17058884 | 9 | 71527774 | G | T | 0.045005 | -0.03183 | 0.005808 | 4.27E-08 | 30.02205 |
| rs17085463 | 4 | 65740387 | A | G | 0.317007 | -0.01481 | 0.002598 | 1.19E-08 | 32.50863 |
| rs17149254 | 7 | 76637391 | C | T | 0.808564 | -0.02381 | 0.003116 | 2.17E-14 | 58.37463 |
| rs17342242 | 8 | 60828697 | G | A | 0.229795 | -0.01616 | 0.002866 | 1.71E-08 | 31.8055 |
| rs17399739 | 10 | 87490850 | G | A | 0.069526 | 0.026237 | 0.004722 | 2.76E-08 | 30.86799 |
| rs17716502 | 8 | 116659731 | T | C | 0.207243 | -0.0223 | 0.002986 | 8.16E-14 | 55.77204 |
| rs17731998 | 3 | 82669493 | T | C | 0.269878 | 0.016159 | 0.002703 | 2.26E-09 | 35.73554 |
| rs1788808 | 18 | 21090023 | G | A | 0.497341 | -0.01831 | 0.002405 | 2.67E-14 | 57.9653 |
| rs1805123 | 7 | 150647969 | G | T | 0.246217 | -0.01799 | 0.002788 | 1.11E-10 | 41.6287 |
| rs1884897 | 20 | 6614691 | G | A | 0.628254 | 0.021329 | 0.002492 | 1.15E-17 | 73.23943 |
| rs1901241 | 12 | 97942845 | G | A | 0.160137 | 0.019292 | 0.00329 | 4.54E-09 | 34.38017 |
| rs1919243 | 5 | 88778861 | C | T | 0.487067 | 0.013873 | 0.002433 | 1.19E-08 | 32.50127 |
| rs1941706 | 18 | 31223776 | G | A | 0.463511 | 0.013941 | 0.002411 | 7.36E-09 | 33.43948 |
| rs1949204 | 13 | 65475834 | G | T | 0.761765 | 0.016653 | 0.002819 | 3.50E-09 | 34.8872 |
| rs2035806 | 10 | 133984916 | A | G | 0.566354 | -0.01663 | 0.002428 | 7.47E-12 | 46.9039 |
| rs2046002 | 15 | 95277910 | C | T | 0.639044 | -0.01457 | 0.002508 | 6.24E-09 | 33.76007 |
| rs2121058 | 13 | 58627256 | C | T | 0.228644 | -0.02381 | 0.002861 | 8.49E-17 | 69.29907 |
| rs2135745 | 9 | 109150784 | G | C | 0.753229 | -0.01684 | 0.002801 | 1.85E-09 | 36.13052 |
| rs2155869 | 18 | 13199302 | C | T | 0.819163 | -0.01845 | 0.003111 | 3.06E-09 | 35.14722 |
| rs215634 | 7 | 32369148 | G | A | 0.613873 | -0.01477 | 0.002476 | 2.45E-09 | 35.57906 |
| rs217672 | 14 | 62361021 | C | A | 0.272013 | 0.015866 | 0.002706 | 4.52E-09 | 34.38773 |
| rs2192649 | 7 | 78126279 | G | T | 0.497183 | 0.013669 | 0.002416 | 1.54E-08 | 32.00719 |
| rs2234458 | 11 | 65639374 | T | C | 0.639151 | -0.02064 | 0.002496 | 1.35E-16 | 68.3868 |
| rs2253310 | 6 | 108888593 | G | C | 0.628085 | 0.017295 | 0.002485 | 3.40E-12 | 48.44748 |
| rs2292238 | 12 | 56493822 | C | A | 0.408667 | -0.0177 | 0.002448 | 4.83E-13 | 52.27931 |
| rs2307111 | 5 | 75003678 | C | T | 0.393183 | -0.02895 | 0.002462 | 6.35E-32 | 138.3022 |
| rs2318543 | 4 | 67803263 | G | A | 0.782495 | -0.0194 | 0.002922 | 3.12E-11 | 44.10116 |
| rs2384054 | 2 | 25156773 | C | T | 0.489674 | 0.035027 | 0.0024 | 3.18E-48 | 212.9834 |
| rs2398861 | 9 | 96430747 | G | A | 0.256599 | 0.022089 | 0.002761 | 1.25E-15 | 63.98957 |
| rs241460 | 1 | 49782772 | G | A | 0.68187 | -0.02054 | 0.002575 | 1.52E-15 | 63.60794 |
| rs2425857 | 20 | 44914134 | G | A | 0.554635 | -0.01359 | 0.002416 | 1.84E-08 | 31.65622 |
| rs2439823 | 10 | 99778226 | G | A | 0.547705 | 0.021636 | 0.002419 | 3.81E-19 | 79.97574 |
| rs2450445 | 10 | 93038530 | A | G | 0.323973 | -0.01537 | 0.002563 | 2.02E-09 | 35.95263 |
| rs245775 | 5 | 170535815 | G | A | 0.728826 | 0.02033 | 0.002705 | 5.65E-14 | 56.49232 |
| rs2470392 | 12 | 2155430 | C | T | 0.287428 | 0.014545 | 0.00266 | 4.54E-08 | 29.90592 |
| rs2474898 | 6 | 51769245 | T | C | 0.345803 | 0.01461 | 0.002523 | 7.03E-09 | 33.52929 |
| rs2482704 | 9 | 94182363 | T | G | 0.423422 | -0.01383 | 0.002432 | 1.30E-08 | 32.32776 |
| rs2606228 | 3 | 183537759 | C | A | 0.642769 | -0.01557 | 0.002536 | 8.29E-10 | 37.69253 |
| rs2609181 | 2 | 6161899 | C | G | 0.281859 | -0.01612 | 0.002687 | 2.00E-09 | 35.97375 |
| rs2616192 | 8 | 20668624 | T | G | 0.67249 | 0.014076 | 0.002572 | 4.45E-08 | 29.94472 |
| rs2678204 | 1 | 201800511 | G | T | 0.341813 | 0.028087 | 0.002536 | 1.65E-28 | 122.6865 |
| rs2711111 | 7 | 24529055 | G | A | 0.565043 | -0.01472 | 0.002457 | 2.12E-09 | 35.86125 |
| rs2725371 | 8 | 30854033 | G | A | 0.696308 | -0.01809 | 0.002621 | 5.12E-12 | 47.64353 |
| rs273505 | 19 | 18217147 | C | T | 0.419906 | 0.018863 | 0.002434 | 9.27E-15 | 60.05035 |
| rs28366156 | 6 | 31671498 | C | T | 0.131356 | -0.029 | 0.003556 | 3.46E-16 | 66.52774 |
| rs28447555 | 3 | 107385668 | T | C | 0.180987 | 0.018508 | 0.003128 | 3.29E-09 | 35.0084 |
| rs28489620 | 22 | 41804716 | A | G | 0.290982 | -0.01473 | 0.002663 | 3.18E-08 | 30.59792 |
| rs2861685 | 2 | 67837553 | C | T | 0.411371 | -0.01765 | 0.002438 | 4.52E-13 | 52.40904 |
| rs286818 | 5 | 107433446 | A | T | 0.170248 | -0.02915 | 0.003204 | 9.41E-20 | 82.73875 |
| rs2962082 | 16 | 62816628 | A | G | 0.482549 | -0.01354 | 0.002408 | 1.88E-08 | 31.61861 |
| rs2975693 | 8 | 10114474 | C | T | 0.109281 | 0.023826 | 0.003875 | 7.85E-10 | 37.79872 |
| rs34045288 | 6 | 40369081 | T | C | 0.335903 | 0.025998 | 0.002545 | 1.72E-24 | 104.3422 |
| rs34236292 | 4 | 140786038 | T | G | 0.32772 | -0.0141 | 0.002567 | 3.96E-08 | 30.16949 |
| rs34361149 | 1 | 72750470 | A | G | 0.19259 | -0.03099 | 0.003045 | 2.50E-24 | 103.5942 |
| rs34373881 | 3 | 20432033 | A | G | 0.278276 | -0.01689 | 0.002687 | 3.28E-10 | 39.5007 |
| rs34774377 | 5 | 167001890 | C | T | 0.120337 | -0.02084 | 0.003691 | 1.63E-08 | 31.88977 |
| rs34811474 | 4 | 25408838 | A | G | 0.231875 | -0.0306 | 0.002843 | 5.30E-27 | 115.8022 |
| rs34966008 | 17 | 34938437 | T | C | 0.408148 | -0.01992 | 0.002443 | 3.59E-16 | 66.45494 |
| rs35025195 | 11 | 134522597 | A | G | 0.166132 | -0.02305 | 0.00323 | 9.68E-13 | 50.91224 |
| rs35193668 | 13 | 33092929 | T | C | 0.361915 | -0.01668 | 0.0025 | 2.55E-11 | 44.49548 |
| rs35483388 | 11 | 122545146 | T | C | 0.379617 | 0.01408 | 0.002486 | 1.48E-08 | 32.07834 |
| rs35626515 | 16 | 28649651 | A | C | 0.407965 | 0.02657 | 0.002445 | 1.71E-27 | 118.0471 |
| rs35722922 | 2 | 47002226 | G | A | 0.385007 | -0.01646 | 0.002478 | 3.08E-11 | 44.12615 |
| rs357501 | 3 | 153953440 | A | G | 0.377514 | 0.015404 | 0.002491 | 6.24E-10 | 38.24613 |
| rs35851183 | 4 | 80717182 | G | A | 0.357777 | 0.016761 | 0.002509 | 2.36E-11 | 44.6463 |
| rs35882248 | 2 | 230627955 | T | C | 0.314642 | 0.019232 | 0.002589 | 1.11E-13 | 55.1585 |
| rs36007635 | 6 | 163009335 | A | G | 0.138403 | -0.02 | 0.003481 | 9.21E-09 | 33.00309 |
| rs362307 | 4 | 3241845 | T | C | 0.076028 | 0.031354 | 0.004582 | 7.74E-12 | 46.83267 |
| rs3759584 | 14 | 103990799 | C | T | 0.361958 | -0.01658 | 0.002514 | 4.20E-11 | 43.51903 |
| rs3803286 | 14 | 103261300 | G | A | 0.666379 | -0.02079 | 0.002545 | 3.08E-16 | 66.75638 |
| rs3810291 | 19 | 47569003 | A | G | 0.677478 | 0.029685 | 0.002568 | 6.54E-31 | 133.6707 |
| rs3843540 | 7 | 99126640 | C | T | 0.148581 | -0.02417 | 0.003382 | 8.89E-13 | 51.07883 |
| rs3844598 | 5 | 140992235 | G | A | 0.528663 | 0.013699 | 0.00241 | 1.32E-08 | 32.307 |
| rs3861879 | 9 | 129464856 | G | A | 0.43683 | 0.013735 | 0.002431 | 1.62E-08 | 31.91142 |
| rs3897102 | 12 | 123492112 | T | C | 0.412248 | 0.014726 | 0.00246 | 2.14E-09 | 35.84765 |
| rs390192 | 6 | 83359135 | G | A | 0.522445 | -0.01544 | 0.002421 | 1.78E-10 | 40.69308 |
| rs4246657 | 2 | 229006134 | T | C | 0.340914 | 0.017006 | 0.002534 | 1.94E-11 | 45.02967 |
| rs4261944 | 4 | 31003636 | G | T | 0.362956 | 0.015489 | 0.002504 | 6.22E-10 | 38.25237 |
| rs4402589 | 16 | 29954654 | G | T | 0.55278 | 0.029152 | 0.002417 | 1.68E-33 | 145.5159 |
| rs4467770 | 6 | 12086826 | A | G | 0.730668 | 0.015669 | 0.002717 | 8.07E-09 | 33.26069 |
| rs4474229 | 1 | 195037001 | A | G | 0.372338 | -0.01588 | 0.002483 | 1.62E-10 | 40.87821 |
| rs4482463 | 2 | 205375909 | A | C | 0.924181 | -0.03409 | 0.004545 | 6.43E-14 | 56.23919 |
| rs4502882 | 5 | 153093998 | T | C | 0.656893 | -0.01472 | 0.002529 | 5.91E-09 | 33.86659 |
| rs4595495 | 10 | 53673286 | G | A | 0.422607 | 0.013738 | 0.002436 | 1.70E-08 | 31.81708 |
| rs4648450 | 1 | 2723214 | A | C | 0.466133 | -0.01544 | 0.002422 | 1.85E-10 | 40.62193 |
| rs4671328 | 2 | 58935282 | G | T | 0.552555 | -0.02177 | 0.002435 | 3.84E-19 | 79.959 |
| rs4687770 | 3 | 51755065 | C | T | 0.133375 | -0.01934 | 0.003535 | 4.50E-08 | 29.9205 |
| rs4718964 | 7 | 70045941 | T | G | 0.412194 | 0.014565 | 0.002447 | 2.64E-09 | 35.43606 |
| rs4757144 | 11 | 13331226 | A | G | 0.593047 | 0.016524 | 0.002446 | 1.42E-11 | 45.64264 |
| rs4776970 | 15 | 68080886 | T | A | 0.35707 | -0.02546 | 0.002505 | 2.89E-24 | 103.3114 |
| rs4777541 | 15 | 73082240 | T | C | 0.765041 | 0.019651 | 0.002846 | 5.08E-12 | 47.65947 |
| rs4778918 | 15 | 79434040 | C | T | 0.422703 | -0.0152 | 0.002432 | 4.11E-10 | 39.0643 |
| rs4790841 | 17 | 1835482 | T | C | 0.154867 | -0.02952 | 0.003329 | 7.49E-19 | 78.63931 |
| rs487662 | 11 | 64481880 | A | G | 0.753235 | 0.015437 | 0.00279 | 3.14E-08 | 30.6222 |
| rs4911382 | 20 | 32553095 | T | C | 0.584238 | 0.015559 | 0.002443 | 1.90E-10 | 40.56604 |
| rs491711 | 11 | 28742220 | C | A | 0.310988 | -0.01584 | 0.002609 | 1.28E-09 | 36.85153 |
| rs4921301 | 5 | 159984492 | T | C | 0.209817 | -0.01822 | 0.002984 | 1.02E-09 | 37.29505 |
| rs4929923 | 11 | 8639200 | C | T | 0.647328 | 0.017384 | 0.002511 | 4.39E-12 | 47.94427 |
| rs525101 | 13 | 111969728 | C | T | 0.371963 | 0.016496 | 0.00249 | 3.50E-11 | 43.87695 |
| rs539515 | 1 | 177889025 | C | A | 0.207529 | 0.047487 | 0.00296 | 6.64E-58 | 257.402 |
| rs550974 | 11 | 118937106 | T | A | 0.404297 | 0.01589 | 0.002448 | 8.55E-11 | 42.13159 |
| rs55689274 | 15 | 47758909 | A | G | 0.285711 | -0.01483 | 0.002663 | 2.53E-08 | 31.0378 |
| rs55726687 | 12 | 991306 | A | G | 0.211138 | 0.023863 | 0.002942 | 5.09E-16 | 65.76938 |
| rs55886426 | 20 | 62567684 | G | C | 0.055432 | -0.03164 | 0.005519 | 9.86E-09 | 32.87097 |
| rs55938344 | 12 | 108310327 | C | A | 0.242702 | -0.01682 | 0.002818 | 2.39E-09 | 35.62593 |
| rs56067609 | 7 | 133582465 | T | A | 0.136594 | -0.01936 | 0.003502 | 3.21E-08 | 30.57519 |
| rs56161855 | 17 | 46288649 | T | A | 0.132008 | 0.022972 | 0.003549 | 9.65E-11 | 41.89398 |
| rs56212061 | 19 | 19394640 | T | C | 0.149969 | -0.02111 | 0.003373 | 3.89E-10 | 39.16929 |
| rs56773984 | 2 | 220177285 | T | A | 0.16742 | -0.01792 | 0.003232 | 2.96E-08 | 30.73754 |
| rs56803094 | 15 | 99222509 | G | A | 0.227111 | -0.01888 | 0.002874 | 5.04E-11 | 43.16273 |
| rs57636386 | 18 | 58048295 | C | T | 0.083415 | -0.04277 | 0.004353 | 8.92E-23 | 96.51386 |
| rs58862095 | 7 | 75081418 | T | C | 0.419757 | -0.02438 | 0.00244 | 1.65E-23 | 99.86151 |
| rs588660 | 1 | 96886604 | A | G | 0.585856 | 0.018301 | 0.002441 | 6.52E-14 | 56.2136 |
| rs59104534 | 8 | 25666169 | T | C | 0.301112 | 0.014978 | 0.002629 | 1.22E-08 | 32.45848 |
| rs5995843 | 22 | 40697377 | G | A | 0.348993 | -0.01744 | 0.002519 | 4.43E-12 | 47.92899 |
| rs6050446 | 20 | 25195509 | G | A | 0.966803 | 0.042077 | 0.00676 | 4.83E-10 | 38.74574 |
| rs60654199 | 3 | 141280448 | A | C | 0.066648 | 0.031266 | 0.00481 | 8.03E-11 | 42.25361 |
| rs60764613 | 18 | 1839911 | T | G | 0.144857 | 0.023587 | 0.003426 | 5.84E-12 | 47.38672 |
| rs61813324 | 1 | 156049877 | T | C | 0.134883 | 0.027919 | 0.003563 | 4.69E-15 | 61.39381 |
| rs61826867 | 1 | 174377435 | G | A | 0.109772 | 0.025135 | 0.003842 | 6.05E-11 | 42.80582 |
| rs61871615 | 10 | 102487140 | T | C | 0.090523 | -0.02803 | 0.004373 | 1.47E-10 | 41.06813 |
| rs61903695 | 11 | 89922417 | G | A | 0.255999 | 0.016359 | 0.002752 | 2.78E-09 | 35.33352 |
| rs61969510 | 13 | 86484025 | C | T | 0.284412 | 0.015533 | 0.002695 | 8.25E-09 | 33.21766 |
| rs62106258 | 2 | 417167 | C | T | 0.048467 | -0.09069 | 0.005592 | 3.93E-59 | 263.0419 |
| rs62147189 | 2 | 86812549 | G | T | 0.622 | -0.01701 | 0.002513 | 1.32E-11 | 45.79252 |
| rs62246314 | 3 | 9504099 | A | G | 0.101276 | 0.0228 | 0.003981 | 1.02E-08 | 32.80709 |
| rs62407562 | 6 | 33530346 | A | T | 0.268967 | 0.017085 | 0.002709 | 2.87E-10 | 39.76183 |
| rs62543438 | 9 | 73896870 | C | G | 0.271966 | -0.0148 | 0.0027 | 4.22E-08 | 30.04781 |
| rs6265 | 11 | 27679916 | T | C | 0.189726 | -0.04023 | 0.003062 | 2.04E-39 | 172.6081 |
| rs6536575 | 4 | 162091639 | C | T | 0.518496 | 0.014062 | 0.002405 | 4.97E-09 | 34.20166 |
| rs6575340 | 14 | 94023972 | A | G | 0.637416 | 0.022878 | 0.002504 | 6.48E-20 | 83.4762 |
| rs6601527 | 8 | 10665444 | A | C | 0.587819 | -0.02174 | 0.002445 | 6.18E-19 | 79.01937 |
| rs66679256 | 4 | 18351898 | T | C | 0.446142 | 0.016297 | 0.002422 | 1.70E-11 | 45.29065 |
| rs6687953 | 1 | 112293512 | G | A | 0.391351 | 0.015895 | 0.002461 | 1.06E-10 | 41.70321 |
| rs66922415 | 18 | 57848651 | G | A | 0.234362 | 0.052096 | 0.002832 | 1.55E-75 | 338.4007 |
| rs6705567 | 2 | 55323183 | C | T | 0.3788 | -0.01362 | 0.002495 | 4.79E-08 | 29.80145 |
| rs6722241 | 2 | 100803778 | C | T | 0.270908 | -0.02017 | 0.002714 | 1.06E-13 | 55.25462 |
| rs6739755 | 2 | 59330227 | G | A | 0.602976 | -0.02096 | 0.00246 | 1.62E-17 | 72.56405 |
| rs67609008 | 10 | 126640936 | C | T | 0.283597 | 0.014601 | 0.002674 | 4.78E-08 | 29.80612 |
| rs6780459 | 3 | 104624105 | T | A | 0.746681 | 0.01636 | 0.002762 | 3.18E-09 | 35.07515 |
| rs67844506 | 20 | 51103807 | G | A | 0.183242 | -0.02592 | 0.003109 | 7.65E-17 | 69.50451 |
| rs6789488 | 3 | 136146030 | C | T | 0.750784 | 0.020009 | 0.002777 | 5.85E-13 | 51.90174 |
| rs6809307 | 3 | 156872076 | T | C | 0.25622 | 0.015707 | 0.00276 | 1.27E-08 | 32.3799 |
| rs6831020 | 4 | 55500226 | A | C | 0.295229 | -0.01594 | 0.002631 | 1.39E-09 | 36.69156 |
| rs6861649 | 5 | 50864788 | C | T | 0.607485 | 0.014348 | 0.002469 | 6.23E-09 | 33.76147 |
| rs6950388 | 7 | 1270699 | A | G | 0.795004 | 0.017228 | 0.002976 | 7.10E-09 | 33.50928 |
| rs7006178 | 8 | 14252384 | C | G | 0.287875 | 0.015504 | 0.002659 | 5.50E-09 | 34.00566 |
| rs7030732 | 9 | 124630006 | A | C | 0.607889 | -0.01516 | 0.002459 | 7.04E-10 | 38.01044 |
| rs704061 | 12 | 89771903 | C | T | 0.452703 | 0.01587 | 0.002414 | 4.88E-11 | 43.22553 |
| rs7094644 | 10 | 16773864 | A | G | 0.674176 | 0.015282 | 0.002611 | 4.81E-09 | 34.26774 |
| rs7116641 | 11 | 43696917 | G | T | 0.316797 | 0.024422 | 0.002586 | 3.67E-21 | 89.1553 |
| rs7124681 | 11 | 47529947 | A | C | 0.408057 | 0.026746 | 0.00244 | 5.90E-28 | 120.1587 |
| rs7132908 | 12 | 50263148 | A | G | 0.38392 | 0.028534 | 0.002472 | 8.27E-31 | 133.203 |
| rs7138383 | 12 | 103724090 | A | G | 0.253485 | -0.02138 | 0.002765 | 1.05E-14 | 59.79833 |
| rs7141420 | 14 | 79899454 | T | C | 0.514078 | 0.020815 | 0.002419 | 7.74E-18 | 74.02761 |
| rs71495049 | 10 | 34014435 | A | G | 0.084031 | 0.02655 | 0.00433 | 8.69E-10 | 37.60176 |
| rs7183417 | 15 | 46586088 | T | C | 0.432569 | 0.0149 | 0.002428 | 8.37E-10 | 37.67348 |
| rs7189149 | 16 | 69144151 | G | C | 0.127107 | 0.021347 | 0.003608 | 3.29E-09 | 35.00597 |
| rs7195386 | 16 | 24578458 | C | T | 0.506441 | -0.01588 | 0.002407 | 4.21E-11 | 43.51668 |
| rs7201895 | 16 | 407723 | A | G | 0.355984 | -0.01734 | 0.002525 | 6.54E-12 | 47.16216 |
| rs7218014 | 17 | 65832016 | C | T | 0.196595 | 0.020699 | 0.003027 | 8.05E-12 | 46.7567 |
| rs72697614 | 1 | 107514107 | A | C | 0.320396 | 0.014951 | 0.002606 | 9.67E-09 | 32.90827 |
| rs72820274 | 2 | 104420858 | A | G | 0.41876 | 0.016002 | 0.002439 | 5.39E-11 | 43.03376 |
| rs72892910 | 6 | 50816887 | T | G | 0.170442 | 0.040166 | 0.003204 | 4.89E-36 | 157.1294 |
| rs72976986 | 19 | 4050424 | A | G | 0.191187 | -0.02405 | 0.003088 | 6.81E-15 | 60.65822 |
| rs73050254 | 7 | 3129614 | A | G | 0.136682 | 0.019322 | 0.003502 | 3.45E-08 | 30.43995 |
| rs73144053 | 3 | 88085826 | A | C | 0.329919 | -0.01536 | 0.00256 | 2.01E-09 | 35.96692 |
| rs73169730 | 3 | 170734438 | G | A | 0.276291 | 0.019463 | 0.002687 | 4.35E-13 | 52.4819 |
| rs7321331 | 13 | 31015138 | A | G | 0.741132 | 0.017555 | 0.002755 | 1.87E-10 | 40.59735 |
| rs73213484 | 4 | 28489339 | T | A | 0.139056 | -0.0212 | 0.003474 | 1.05E-09 | 37.22678 |
| rs7331420 | 13 | 99236471 | A | G | 0.284027 | -0.01473 | 0.00267 | 3.44E-08 | 30.44309 |
| rs7442885 | 5 | 87682877 | G | C | 0.210104 | -0.02543 | 0.002957 | 7.96E-18 | 73.97136 |
| rs7498044 | 15 | 92573639 | A | G | 0.218661 | -0.01765 | 0.002932 | 1.75E-09 | 36.23293 |
| rs750090 | 4 | 152931436 | C | T | 0.356153 | -0.01563 | 0.002535 | 6.93E-10 | 38.04123 |
| rs752179 | 3 | 108024070 | A | T | 0.301702 | -0.01437 | 0.002621 | 4.17E-08 | 30.07006 |
| rs75499503 | 6 | 26145217 | T | C | 0.219955 | -0.01978 | 0.002946 | 1.88E-11 | 45.09473 |
| rs7553158 | 1 | 75005238 | A | G | 0.562389 | -0.01742 | 0.002424 | 6.70E-13 | 51.63391 |
| rs75557510 | 3 | 84321469 | G | A | 0.061496 | -0.03779 | 0.005104 | 1.33E-13 | 54.81237 |
| rs756717 | 16 | 72996162 | A | G | 0.398859 | -0.01467 | 0.002482 | 3.38E-09 | 34.95425 |
| rs76040172 | 21 | 46488959 | A | G | 0.05444 | -0.04076 | 0.005308 | 1.62E-14 | 58.95047 |
| rs7701777 | 5 | 27178172 | G | T | 0.282759 | -0.01703 | 0.002668 | 1.74E-10 | 40.74041 |
| rs7707394 | 5 | 74472939 | A | G | 0.354316 | -0.02019 | 0.002507 | 8.12E-16 | 64.84818 |
| rs7719067 | 5 | 153538241 | G | A | 0.573835 | -0.0162 | 0.002426 | 2.41E-11 | 44.60636 |
| rs7723426 | 5 | 43186097 | C | T | 0.675017 | 0.014669 | 0.002566 | 1.09E-08 | 32.68151 |
| rs7755574 | 6 | 153365100 | T | G | 0.281153 | 0.015496 | 0.002674 | 6.87E-09 | 33.57304 |
| rs7774 | 17 | 4801163 | A | C | 0.309138 | 0.016634 | 0.002615 | 2.01E-10 | 40.45518 |
| rs778094 | 2 | 147903802 | A | G | 0.577568 | -0.01485 | 0.002437 | 1.09E-09 | 37.15749 |
| rs7852189 | 9 | 103121547 | G | A | 0.311364 | 0.016554 | 0.002589 | 1.61E-10 | 40.88842 |
| rs78565420 | 8 | 85703065 | T | C | 0.054186 | 0.033731 | 0.005454 | 6.24E-10 | 38.24627 |
| rs79113395 | 1 | 1590521 | A | G | 0.265449 | -0.02091 | 0.002732 | 1.95E-14 | 58.59096 |
| rs7933085 | 11 | 130796248 | G | A | 0.509195 | 0.015739 | 0.002415 | 7.21E-11 | 42.46469 |
| rs7952102 | 11 | 892089 | C | T | 0.387339 | -0.01523 | 0.002465 | 6.59E-10 | 38.14076 |
| rs7992832 | 13 | 28013501 | T | C | 0.276544 | -0.01756 | 0.002692 | 7.02E-11 | 42.51724 |
| rs799449 | 7 | 44784697 | T | C | 0.558481 | 0.019902 | 0.002427 | 2.41E-16 | 67.24461 |
| rs8015400 | 14 | 25930988 | A | C | 0.678151 | 0.021088 | 0.002572 | 2.45E-16 | 67.21029 |
| rs80330591 | 2 | 159368720 | A | G | 0.146844 | -0.02136 | 0.003393 | 3.08E-10 | 39.62414 |
| rs8078135 | 17 | 21268583 | T | C | 0.488467 | -0.0195 | 0.002412 | 6.23E-16 | 65.36947 |
| rs8087074 | 18 | 45923482 | T | G | 0.261119 | 0.017007 | 0.002748 | 6.02E-10 | 38.31584 |
| rs8134638 | 21 | 40644170 | C | T | 0.375763 | 0.015169 | 0.002481 | 9.68E-10 | 37.39042 |
| rs815163 | 1 | 190294726 | C | T | 0.561603 | -0.01825 | 0.00242 | 4.69E-14 | 56.85962 |
| rs845084 | 10 | 125220036 | A | G | 0.257551 | 0.02034 | 0.002757 | 1.61E-13 | 54.4327 |
| rs862320 | 16 | 69651866 | T | C | 0.410152 | -0.02282 | 0.002446 | 1.10E-20 | 86.98576 |
| rs869400 | 3 | 185826740 | G | T | 0.815827 | 0.030726 | 0.003107 | 4.70E-23 | 97.78402 |
| rs879620 | 16 | 4015729 | T | C | 0.615276 | 0.025612 | 0.002478 | 4.93E-25 | 106.8148 |
| rs9267671 | 6 | 31880480 | A | G | 0.060402 | 0.032371 | 0.005042 | 1.36E-10 | 41.22704 |
| rs9291822 | 5 | 64077647 | T | C | 0.515455 | -0.01361 | 0.002426 | 2.00E-08 | 31.49937 |
| rs9320823 | 6 | 98429337 | C | T | 0.601901 | 0.01894 | 0.002458 | 1.30E-14 | 59.39254 |
| rs9342196 | 6 | 90124635 | T | C | 0.189501 | 0.016733 | 0.003066 | 4.83E-08 | 29.78377 |
| rs935166 | 2 | 26949366 | A | G | 0.506935 | -0.01549 | 0.002403 | 1.13E-10 | 41.58162 |
| rs9402104 | 6 | 129410736 | A | G | 0.584134 | 0.013902 | 0.002452 | 1.43E-08 | 32.14605 |
| rs946185 | 10 | 76423739 | G | A | 0.593276 | -0.01469 | 0.002462 | 2.41E-09 | 35.61381 |
| rs9515455 | 13 | 112239671 | A | G | 0.416606 | 0.017964 | 0.00245 | 2.25E-13 | 53.7767 |
| rs9527906 | 13 | 59403551 | A | G | 0.762395 | -0.01618 | 0.002834 | 1.13E-08 | 32.60992 |
| rs9641499 | 7 | 112984493 | A | C | 0.434095 | -0.0171 | 0.002423 | 1.68E-12 | 49.83371 |
| rs9688977 | 6 | 154336892 | C | T | 0.146486 | 0.023978 | 0.003406 | 1.93E-12 | 49.55796 |
| rs9835772 | 3 | 85766025 | T | A | 0.242763 | 0.019651 | 0.0028 | 2.24E-12 | 49.26584 |
| rs9843653 | 3 | 49920571 | C | T | 0.514555 | 0.031726 | 0.002404 | 9.35E-40 | 174.1592 |
| rs9847186 | 3 | 25081857 | A | G | 0.427737 | -0.01428 | 0.002434 | 4.36E-09 | 34.45813 |
